# Method of oral delivery affects vitamin C-mediated alleviation of colitis in a mouse model

**DOI:** 10.1080/29933935.2025.2549734

**Published:** 2025-09-18

**Authors:** Pi Westi Bondegaard, Katja Ann Kristensen, Khorshid Kamguyan, Jette Jakobsen, Vanessa Emily Rees, Mahdi Ghavami, Line Hagner Nielsen, Anja Boisen, Martin Iain Bahl, Tine Rask Licht, Martin Steen Mortensen

**Affiliations:** a National Food Institute, Technical University of Denmark, Lyngby, Denmark; b Department of Health Technology, Technical University of Denmark, Lyngby, Denmark; c The Novo Nordisk Foundation Center for Biosustainability, Technical University of Denmark, Lyngby, Denmark

**Keywords:** Vitamin C, antioxidant, ulcerative colitis, DSS mouse model, gut microbiota, microcontainers

## Abstract

Vitamin C could be a potential candidate for antioxidant therapy in ulcerative colitis (UC) patients, but efficient small intestinal absorption of vitamin C prevents it from reaching the colon, limiting the direct antioxidative effect on inflamed colonic tissue. We applied a colitis mouse model to compare three oral delivery strategies for high-dose vitamin C: dissolved in drinking water, mixed into peanut butter, and in colon-targeted microcontainers administered with peanut butter. Mice were administered dextran sulfate sodium (DSS) to induce UC symptoms. Healthy control and DSS control groups were included. Alleviation of disease symptoms and microbial dysbiosis were assessed to elucidate the effects of encapsulation and stabilization of vitamin C during intestinal transit. Vitamin C in peanut butter, independent of microcontainers, alleviated UC symptoms. Administration in drinking water surprisingly exacerbated symptoms compared to the DSS control group. DSS-treatment caused an increased level of facultative anaerobes in the fecal microbial community that was not affected by vitamin C administration. Vitamin C partially reduces disease activity index in DSS-treated mice, when delivered in peanut butter, likely promoting vitamin C stability. Drinking water delivery did not support vitamin C-related alleviating effects. None of the evaluated delivery strategies affected the DSS-induced changes of the microbial community.

## Introduction

Ulcerative colitis (UC) prevalence is increasing worldwide; it impairs quality of life, with common symptoms including fecal abnormalities, bloody diarrhea, and abdominal pain. UC is a relapsing, chronic disease, manifested by inflammation in the colon and rectum.^1,2^ Although several therapeutic strategies have been developed, they have undesired side-effects and low efficacy, and alternative treatment approaches are thus needed.^3^ The etiology of UC is a poorly understood complex interplay between genetic and environmental factors, the intestinal microbiota, and the immune system.^2^ Furthermore, oxidative stress and depletion of antioxidants are considered key factors in disease development and progression.^4^ Oxidative stress occurs when an elevated production of reactive oxygen species (ROS), exceeds the antioxidant defense mechanisms’ capacity, and may lead to tissue damage and intensification of destructive immune responses.^3,5^ Extensive epithelial repair and pro-inflammatory signals change colonocyte metabolism, leading to increased amounts of oxygen (O_2_) in the intestinal environment.^6^ In a healthy state, butyrate fermented from fibers by strict anaerobic bacteria is converted to CO_2_ by β-oxidation under consumption of O_2_, ensuring low levels of O_2_ in the colonic epithelium and lumen. However, when this is disrupted in UC, colonocyte metabolism is dominated by anaerobic glycolysis, which does not consume O_2_.^6^ The increased availability of O_2_ promotes expansion of facultative anaerobic bacteria, especially of the family *Enterobacteriaceae* (phylum *Proteobacteria*), and decrease in butyrate-producing strict anaerobic bacteria of the *Firmicutes* phylum, including *Faecalibacterium prausnitzii* – this is a hallmark characteristic of microbial dysbiosis in UC patients.^7–10^

To combat the destructive state of oxidative stress and derived dysbiotic effects, antioxidant therapies are studied as alternative or add-on treatments to present medical strategies. Various compounds have been tested, some showing potential for UC treatment or alleviation.^3,5^ Vitamin C is an effective, water-soluble antioxidant with multiple biological functions, including involvement in enzymatic reactions and reduction of oxidative stress damage through its ROS scavenging properties. Vitamin C deficiency frequently occurs in UC patients, particularly in subjects with elevated inflammatory markers,^11^ and vitamin C levels in biopsies of inflamed tissue are markedly lower compared to healthy tissue.^12^ In a DSS mouse model of UC, significant alleviation of symptoms, reduction of oxidative stress markers and pro-inflammatory immune responses, were observed after intraperitoneal treatment with vitamin C during and after DSS administration.^13^ In line with this, high-dose intraperitoneal vitamin C alleviated DSS-induced UC in mice,^14^ while vitamin C insufficiency in Gulo (-/-) mice, unable to synthesize vitamin C, increased severity of DSS-induced UC.^15^ Taken together, vitamin C is a potential candidate for UC antioxidant therapy. However, it is efficiently absorbed in the small intestine, preventing it from reaching the colon^16^ and limiting the direct antioxidative effects on inflamed colonic tissue, local oxidative stress, and the dysbiotic microbiota in UC patients. Furthermore, vitamin C is very unstable in aqueous solutions when exposed to environmental factors such as increased temperature, light, and the presence of metals.^17^ Encapsulation strategies hold promise to increase stability.^18,19^

In the present study, we aim to increase effect of vitamin C treatment in a mouse model of DSS-induced UC through (i) local delivery of vitamin C to the site of disease, (ii) protection from uptake in the small intestine, and (iii) increased antioxidant stabilization. We compared a delivery system of cylindrical microcontainers^20–22^ embedded in peanut butter, with vitamin C powder directly embedded in peanut butter, or added to the drinking water. A peanut butter delivery strategy was applied,^23,24^ as an alternative to delivery by daily gavage, to encourage voluntary ingestion of a high enough number of microcontainers to reach a relevant vitamin C dosage. We included a healthy control group (no DSS) and a DSS-treated control group, both fed with peanut butter without vitamin C. Faecal microbial composition and activity, as well as selected disease markers were assessed.

## Materials and methods

### Animals and experimental setup

**Figure 1.**
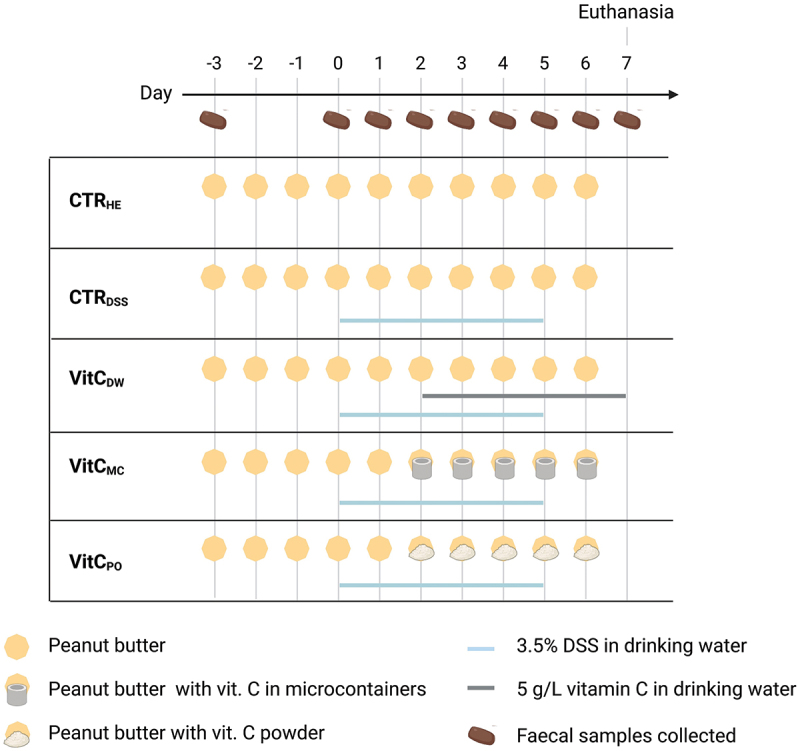
Experimental timeline and dosing of groups. All groups received peanut butter at Days −3 to 6. The healthy controls (CTR_HE_) did not get additional treatment. The DSS controls (CTR_DSS_) was treated with DSS from Day 0 to 5. The vitamin C in drinking water group (VitC_DW_) was treated with DSS from Day 0 to 5, and vitamin C in the drinking water from Day 2 to 7. The vitamin C in microcontainer group (VitC_MC_) was treated with DSS from Day 0 to 5, and vitamin C administered in microcontainers from Day 2 to 6. The vitamin C as powder group (VitC_PO_) was treated with DSS from Day 0 to 5, and vitamin C administered as powder from Day 2 to 6. Faecal samples were collected at Days −3 and 0–7. Created with BioRender.com.

This study was carried out under approval by the Animal Welfare Committee, license number 2020–15–0201–00464 C1 by trained and skilled personnel at the animal facility at the National Food Institute, Technical University of Denmark. The experimental timeline is outlined in Figure 1.

Forty female C57BL/6NTac mice, 7–8 weeks, MPF health status (Taconic Tornbjerg, Ejby, Denmark), were caged in controlled, ventilated cabinets (ScanTainer, Scanbur, Karlslunde, Denmark) with ad libitum access to feed, without vitamin C (Altromin AIN-93 G pellets, Lage, Germany), and drinking water.

On Day −6 all mice were single housed and on Day 0 randomized to five groups with 8 mice in each: Healthy control group. DSS control group, receiving 3.5% DSS in the drinking water (Day 0–5). Vitamin C in drinking water group, receiving 3.5% DSS (Day 0–5) and 5 g/L vitamin C (Day 2–7) in the drinking water. Vitamin C in microcontainers group, receiving 3.5% DSS in the drinking water (Day 0–5) and vitamin C in microcontainers dosed in peanut butter (Day 2–6). Vitamin C as powder group, receiving 3.5% DSS in the drinking water (Day 0–5) and vitamin C as powder dosed in peanut butter (Day 2–6). For the microcontainers and powder groups, the last vitamin C dosing was on Day 6. To account for prolonged release within these two dosing forms, the drinking water group continued to receive vitamin C in the drinking water till the end of study (Day 7).

From Day −3 to Day 1 all mice received 0.1 g/day of peanut butter (Peanut Butter Creamy, Green Choice, Hvidovre, Denmark) as adaption to the dosing procedure, followed by continued administration to Day 6 with pure peanut butter for healthy control, DSS control, and drinking water groups and peanut butter containing vitamin C in microcontainers or as powder for the dosing groups. The peanut butter dosing strategy^23,24^ was done by placing the peanut butter at a defined spot on each animal cage wall from where it was eaten by the mice.

The weight of all animals was followed from Day 0 to 7 and scored by percent weight loss (0: normal; 1: 0–5%; 2: 5–10%; 3: 10–15%; 4: > 15%). In the same period, we also scored fecal texture (0: solid, normal healthy consistency; 2: soft, could be reshaped without cracking; 4: very soft, almost liquid) and fecal blood content (0: no blood; 2: blood, sign of red in the feces; 4: a lot of blood, feces mainly red) for each mouse. The disease activity index (DAI) was calculated as the sum of scores for these three factors.^13^ Fecal samples were collected on Days −3 (before starting peanut butter adaption), 0 (before starting DSS treatment), 1, 2, 3, 4, 5, 6 and 7. Some animals did not deliver fecal samples on Days 5–7. One mouse receiving vitamin C as powder was euthanized on Day 5 due to extensive disease symptoms. The rest of the mice were euthanized and dissected on Day 7.

### Microcontainers

#### Microcontainer fabrication, loading, and coating

In this study, cylindrical microcontainers with an inner diameter of 229 ± 0.1 µm (mean ± SD) and inner height of 210 ± 2.3 µm (mean ± SD) were applied. The devices were designed for unidirectional release and fabricated from SU-8 (SU-82035, 2075, micro resist technology GmbH, Berlin, Germany), through a two-step photolithographic process. The microcontainers were produced on silicon wafers coated with 5 nm Ti and 20 nm Au, and subsequently, cut into chips containing 625 microcontainers each.^25^

Microcontainers were manually loaded by compressing ground vitamin C powder (L-Ascorbic Acid, A92902, Sigma-Aldrich, St. Louis, USA) on top of a microcontainer chip coved by a microfabricated shadow mask. Each chip was loaded with 4.2 ± 0.7 mg (mean ± SD) vitamin C powder. After loading and removal of the mask, the microcontainer chips were coated with 1% w/v Eudragit® FS100 (Evonik, Essen, Germany) in a 90% ethanol solution for 800 passages over the sample using an ultrasonic spray coater (Exactacoat system, Sono-Tek, Milton, NY, USA). The vortex nozzle was operating with an infusion rate of 1.5 mL/min, a generator control of 1.5 W, air pressure of 0.02 bar, nozzle translation speed of 20 mm/s, and the chip was laying on a plate heated to 40°C. An additional layer of coating was applied using a 1% w/v Eudragit® L100 (Evonik, Essen, Germany) in an isopropanol solution, containing 5% w/w (in relation to the polymer) dibutyl sebacate as a plasticizer, for 150 passages over the sample using the same parameters. Both loading and coating were evaluated in a TM3030Plus tabletop scanning electron microscope (SEM, Hitachi High Technologies Europe GmbH, Krefeld, Germany) (Supplementary Figure S1). The coated microcontainers were left at room temperature to dry overnight and were then scraped off the chip using a scalpel.

#### In vitro validation of coating

The release profile of vitamin C from microcontainers with the prepared coating was validated *in vitro* using a Microdiss Profiler™ (Pion, MA, USA) for real-time UV spectrophotometric measures. A stepwise experiment was conducted where release of vitamin C was measured from 3 coated and 3 uncoated microcontainer chips in HEPES buffer (20 mM, pH adjusted to 4.3) for 1 h, followed by measurement in HEPES buffer (20 mM, pH adjusted to 6.5) for 24 h to mimic gastrointestinal transit (Supplementary Figure S2). The temperature was set to 37°C, the stirring rate was 100 rpm and UV probes with a path length of 5 mm were used. A standard curve of vitamin C concentration from 50 to 350 µg/mL was used.

### Vitamin C and DSS preparation and administration

#### DSS in drinking water

3.5 w/v% DSS (Dextran sulfate sodium salt, Mr ~ 40,000, 42867, Sigma-Aldrich, St. Louis, USA) was dissolved in tap water and sterile filtered (500 mL, 0.2 µm aPES membrane, FB12566504, Fisherbrand, Waltham MA, USA).

#### Vitamin C in drinking water

3.5 w/v% DSS (Dextran sulfate sodium salt, Mr ~ 40,000, 42867, Sigma-Aldrich, St. Louis, USA) and 5 g/L vitamin C (L-Ascorbic Acid, A92902, Sigma-Aldrich, St. Louis, USA) were dissolved in tap water, using a magnet stirrer, and sterile filtered (500 mL, 0.2 µm aPES membrane, FB12566504, Fisherbrand, Waltham MA, USA).

For drinking water only containing vitamin C, 5 g/L vitamin C (L-Ascorbic Acid, A92902, Sigma-Aldrich, St. Louis, USA) were mixed into tap water and prepared as described above.

The dose of vitamin C was estimated to be app. 15 mg vitamin C/mouse/day.

#### Vitamin C in microcontainers and powder

A dose of 4.2 ± 0.7 mg (mean ± SD) vitamin C powder/mouse/day (L-Ascorbic Acid, A92902, Sigma-Aldrich, St. Louis, USA) was administered in 625 coated microcontainers (one chip) mixed into 0.1 g of room temperature peanut butter (Peanut Butter Creamy, Green Choice, Hvidovre, Denmark).

In the same way, a dose of app. 4.2 mg ground vitamin C powder/mouse/day (L-Ascorbic Acid, A92902, Sigma-Aldrich, St. Louis, USA) was administered in peanut butter.

### 16S rRNA gene amplicon sequencing

Genomic DNA was extracted from fecal samples collected at Days −3, 0, 1, 2, 3, 4 and 6, and pellets from Days 5 and 7 (1.3–79.2 mg starting material), using the Costar® 96 DNA Stool kit (MACHEREY-NAGEL, Düren, Germany). 30 µL nuclease free water (Qiagen, Hilden, Germany) was used as negative control. Agitation was performed using a Bead Ruptor Elite Bead Mill Homogenizer at 5 m/s for 30 s (OMNI International, Kennesaw, USA). DNA concentrations was subsequently measured using the SpinX® dsDNA HS Assay Kit (zol™, Thermo Fisher Scientific, Waltham, USA). The V3-region of the 16S rRNA gene was amplified in a 2-step PCR reaction using 5 ng DNA as template, 4 µL HF-buffer x5, 0.4 µL dNTP mix (10 mM), 0.2 µL Phusion High-Fidelity DNA Polymerase (F-530 L, Thermo Fisher Scientific., Waltham, USA), 2 µL reverse primer (10 pmol/µL, PBR 5’-trP1-adapter-ATTACCGCGGCTGCTGG-3’) and 2 µL forward primer (10 pmol/µL, PBU 5’-A-adapter-TCAG-barcode-CCTACGGGAGGCAGCAG-3’). The primers (TAG Copenhagen A/S, Copenhagen, Denmark) contained sequencing adaptors, and the forward primer had a 10–12 bp barcode unique for each sample (Ion Xpress^TM^ Barcode Adapters, Thermo Fisher Scientific, Waltham, USA). The following PCR program was used: 98°C for 30 s, (98°C for 15 s, 72°C for 30 s) x30 cycles, 72°C for 5 min. and cooling to 4°C using a VeritiPro 96-Well Thermal Cycler (Applied SYBR®, Thermo Fisher Scientific, Waltham, USA). A negative control of nuclease free water (QIAGEN, Hilden, Germany) and a positive control of ZymoBIOMICS^TM^ Microbial Community DNA Standards (Zymo Research, Irvine, USA) were included in each PCR run. PCR products were purified using the HighPrep^TM^ PCR Magnetic Beads (AC-60250, MagBio Genomics, Gaithersburg, USA) and a 96-well magnet stand (MyMag 96 Magnetic Plate, MagBio Genomics, Gaithersburg, USA) using nuclease free water (QIAGEN, Hilden, Germany) as elution solution. DNA concentrations were measured using the LightCycler® dsDNA HS Assay Kit (NucleoSpin®, Thermo Fisher Scientific., Waltham, USA) as described above and mixed in equimolar libraries. Samples were sequenced on 4 separate chips using an Ion S5^TM^ Semiconductor Sequencer (Thermo Fisher Scientific, Waltham, USA) applying an Ion 520™ Chip Kit (Thermo Fisher Scientific, Waltham, USA).

All samples were randomized (verified by Chi^2^ tests performed in R studios^26^) before DNA extraction and sequencing.

### SCFA analysis

Fecal samples from Days 5 and 7 were analyzed for the concentration of the following short chain fatty acids (SCFAs): Acetic acid, formic acid, propanoic acid, 2-methyl-propanoic acid, butanoic acid, 3-methylbutanoic acid, pentanoic acid, 4-methyl-pentanoic acid, hexanoic acid and heptanoic acid. The fecal samples were diluted 4x wt./vol. in MiliQ water, homogenized and centrifuged (16,000 g/4°C/10 min.). The pellets were stored at −80°C for later DNA purification and 16S rRNA gene amplicon sequencing. The supernatants were filtered (Costar® SpinX® centrifuge filter, 0.22 µm pore nylon membrane, Corning, USA) by centrifugation at 15,000 g/4°C/5 min. and stored at −80°C. The following GC-MS analysis was performed by MS-OMICS (Vedbæk, Denmark).

### End point measurements (Day 7)

#### Colon length

On dissection, cecum and colon were collected and the colon length measured from the cecal attachment point to the rectum.

#### Gene expression of cytokines

Tissue samples from the proximal colon (app. 0.5 cm) were collected, washed in sterile PBS and stored in 1 mL RNAlater™ Soln. (Invitrogen™, Thermo Fisher Scientific, Waltham, USA) at room temperature the first hours, then transferred to 4°C overnight and finally stored at −80°C until analysis of host expression (mRNA) of the following cytokines: TNF-α, IL-1β, IL-6, IL-17 and IL-10.

For each sample, up to 5 mg colonic tissue was homogenized with glass mini-beads (Thermo Fisher Scientific, Waltham, USA) and 300 µL TRI reagent (Merck Life Science, Sigma Aldrich, St. Louis, USA) on a mini-bead beater for 6 min. Samples were centrifuged at 10,000 g for 3 mins. RNA was isolated from the supernatant using Direct-Costar® RNA Miniprep Plus (Zymo Research, Irvine, USA), including a DNase I treatment, following manufacturer’s protocol. The RNA concentration was determined via NanoDrop 1000 spectrophotometer. cDNA was synthesized from 0.5 µg RNA in 20 µL reactions with ThermoFisher High Capacity cDNA kit. Real-time quantitative PCR was performed on the cDNA using 2x SensiFAST SpinX® Lo-ROX mix (Nordic BioSite, Täby, Sweden) on a Roche Costar® 480 Real-time PCR System. Primers were obtained from Primerbank (http://pga.mgh.harvard.edu/primerbank/.) and can be found in Supplementary Table S1. Thermocycler conditions consisted of an initial 3 min denaturing step at 95°C, followed by 40 cycles of 95°C for 10 sec, 60°C for 15 sec, and 72°C for 30 sec, and a melting curve analysis (95°C for 5 sec, 60°C for 1 min and 95°C for 5 sec). The mRNA expression of the target genes was analyzed by the 2-ΔΔCT method and normalized to the endogenous TATA-box-binding protein (Tbp) gene as an internal control.

#### Vitamin C concentration in colon content

Colon content (4.4−88.7 mg) was collected, snap frozen, and stored at −80°C until quantification of vitamin C (sum of ascorbic acid [AA] and Dehydroascorbic acid [DHAA]) as follows: 1 mL extraction solution consisting of 2% meta-phosphoric acid, 0,1% oxalic acid and 20 mM tris (2-carboxyethyl)-phosphine hydrochloride (TCEP) was added, shaken for 10 min. at room temperature, and allowed to settle for 5 min. In a Sarstedt tube (15 ml), 1 ml Milli-Q® water (Merck Millipore, Darmstadt, Germany) was added, the mixed solution transferred to centrifuge tubes with filters, and centrifuged (10 min., 3000 g, 4° C). 5 µl was injected to a UPLC equipped with DAD (Waters, Milford, USA), using 265 nm and a C18-column (Acquity BEH C18 column, 1.7 µm, 100 × 2.1 mm, Waters, Milford, USA). The mobile phase was adjusted to pH 5.4 using a 2.3 mM dodecyltrimethylammonium chloride, 1% acetonitrile, 10% 0.5 M acetate buffer. Quantification was conducted using an external calibration standard of ascorbic acid. Chemicals were from Sigma-Aldrich (St. Louis, USA). Measurements that were determined unreliable (*n* = 10) were removed prior to analysis, leading to smaller group sizes (Figure 3(A)). Furthermore, a very extreme outlier was excluded.

#### Lipocalin-2 in serum

Heart blood from anesthetized mice was collected, incubated for minimum 0.5 h at room temperature and centrifuged for 10 min. at 6000 rpm. The serum was stored at −20°C until analysis using the Lipocalin-2 (NGAL) Mouse SimpleStep ELISA® Kit, ab199083 (Abcam, Cambridge, USA).

### Data analysis and statistics

Statistical analysis and visualization of data was performed in R studios,^26^ using tidyverse,^27^ ggpubr^28^ and rstatix.^29^

#### Statistical analysis on disease markers

For comparison of means between groups, we first tested the assumptions of normality, heterogeneity, and independence. We applied ANOVA or Kruskal-Wallis test, as appropriate, followed by pairwise comparisons using *t*-test or Dunn’s test, respectively. Correction for multiple testing was done using the Benjamin-Hochberg method.^30^

Analysis of Lipocalin-2 levels were performed on log_10_-transformed data.

Statistical analysis of all SCFAs was initially performed by a MANOVA test to indicate which SCFAs to test individually. A non-parametric PERMANOVA was run in parallel since it is more robust to violations of heteroscedasticity. For SCFAs, where differences were significantly explained by treatment we performed simple comparisons of means as described above.

#### Bioinformatic analysis of sequencing data

16S rRNA gene amplicon data was processed via our in-house pipeline^31^ using the default settings. Briefly, raw amplicon sequence data was demultiplexed using cutadapt (v. 4.1),^32^ denoised using DADA2 (v. 1.22)^33^ and ASVs classified against rdp_train_set_18.^34^ Further processing was done using Phyloseq (v.1.42.0)^35^ running in R (v. 4.2).^26^

Statistical tests on microbial data were performed as follows: Alpha diversity (calculated as observed richness, Shannon diversity index and FaithPD) was analyzed as simple comparisons of means. For beta diversity analysis we used the Aitchison distance matrix. PERMANOVA (function adonis2)^36^ was used for overall analysis of beta diversity and post hoc tests within each day. Differential abundances at Day 3, 5 and 7 were analyzed with a Quasi-Poisson GLM test, using the DAtest implementation.^37^

The order *Enterobacterales* was classified as facultative anaerobic bacteria, and their relative abundance was analyzed by a comparison of means.

Statistical significance was set to *p* < 0.05 and represented as * = p < 0.05, ** = p < 0.01, *** = p < 0.001, **** = p < 0.0001 in plots. The exact p-values for all plots are included in Supplementary file 1.

Scripts for complete data analysis are available at https://github.com/MSMortensen/VitC_delivery. and raw fasq files are uploaded to SRA, BioProject ID PRJNA1045461 (http://www.ncbi.nlm.nih.gov/bioproject/1045461).

## Results

### Alleviating effects on body weight and faecal texture

**Figure 2.**
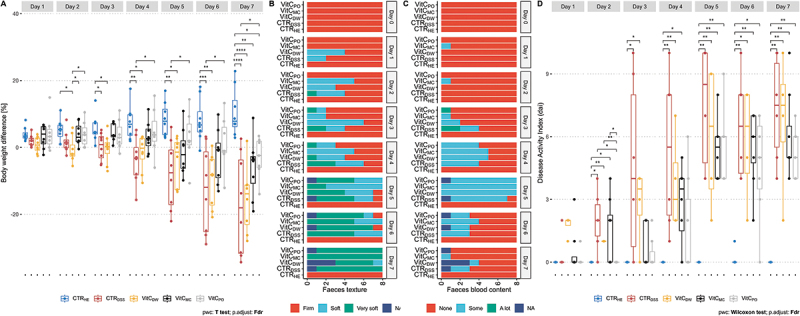
Body weight and faecal scoring. A. Body weight difference [%] normalised to Day 0 for each mouse. Results are shown for Day 1 to 7 for the following groups: CTR_HE_ (blue, *n* = 8), CTR_DSS_ (red, *n* = 8), VitC_DW_ (yellow, *n* = 8), VitC_MC_ (black, *n* = 8) and VitC_PO_ (grey, *n* = 7, one mouse was excluded, since it was euthanised at Day 5). Individual measurements are represented by points in the boxplot. A one-way ANOVA with FDR adjustment was performed within each day, followed by a pairwise *t* test with FDR adjustment between treatment groups for days with interactions found significant. B. Bar plot showing texture of faecal pellets on Day 0 to 7 with scoring of mice presenting firm, soft, very soft or NA (no faeces) [number of mice, *n* = 8]. C. Bar plot showing blood content in faecal pellets on Day 0 to 7 with scoring of mice presenting none, some, a lot or NA (no faeces) [number of mice, *n* = 8]. D. Disease activity index (DAI). Results are shown for Day 1 to 7 for the following groups: healthy controls (CTR_HE_, blue, *n* = 8), DSS controls (CTR_DSS_, red, *n* = 8), the drinking water group (VitC_DW_, yellow, *n* = 8), the microcontainers group (VitC_MC_, black, *n* = 8) and the powder group (VitC_PO_, grey, *n* = 7, one mouse was excluded, since it was euthanised at Day 5). Individual measurements are represented by points in the boxplot. A Kruskal-Wallis test with FDR adjustment was performed within each day, followed by a pairwise Wilcoxon test with FDR adjustment between treatment groups for day 2–7. * = *p* < 0.05, ** = *p* < 0.01, *** = *p* < 0.001, **** = *p* < 0.0001.

Effects of selected delivery strategies for vitamin C on disease markers and the fecal microbiota in a UC mouse model were assessed. Body weights of all mice (Figure 2(A)) as well as fecal texture and content of blood (Figure 2(B,C)) were measured daily from Day 0 to 7 and combined to determine the DAI (Figure 2(D)). Weights of healthy controls (n = 8) increased throughout the period (ANOVA, p = 0.0013). Significant decreases compared to healthy controls were found with an early onset for DSS controls (Day 3, n = 8) and the drinking water group (Day 2, n = 8), whereas the microcontainers (n = 8) and powder (n = 7) groups did not differ significantly from healthy controls until Day 7, even though a decreasing trend was observed before that. Interestingly, the weights for the powder group were significantly higher than for DSS controls from Day 4 to Day 7 (t-test, p.adj < 0.038). Together this indicated an alleviation of disease severity when vitamin C was delivered in peanut butter either in microcontainers or as powder (Figure 2(A)). In line with this, fewer mice within the groups receiving vitamin C in microcontainers or as powder had soft or very soft feces compared to DSS controls and the powder group on Days 1 to 3. However, the alleviating effect was not observed continuously from Day 4 to 7 (Figure 2(B)). Blood content in feces did not reflect any inter-group differences but increased for all groups receiving DSS until end of DSS treatment at Day 5, after which the number of mice presenting fecal blood decreased (Figure 2(C)). The healthy control group (n = 8) was the only group that did not increase in DAI (Figure 2(D)) and there was a significant difference between the groups from day one (Kruskal-Wallis test, p.adj < 0.0135). On day 2 the healthy controls and the powder (n = 7) groups were the only ones where all mice had a DAI score of 0 and the DSS control (n = 6), drinking water (n = 4) and microcontainers (n = 8) groups were significantly higher than both (Wilcoxon-test, p.adj < 0.048). On day 3–7 the DSS controls and the drinking water groups both had significantly higher DAI than the healthy controls (Wilcoxon-test, p.adj < 0.034). Compared to healthy controls the microcontainers group had significantly higher DAI on day 4–7 (Wilcoxon-test, p.adj < 0.0133), while the powder group were significantly higher from day 5 (Wilcoxon-test, p.adj < 0.0133).

### Disease markers and vitamin C concentration in colon

**Figure 3.**
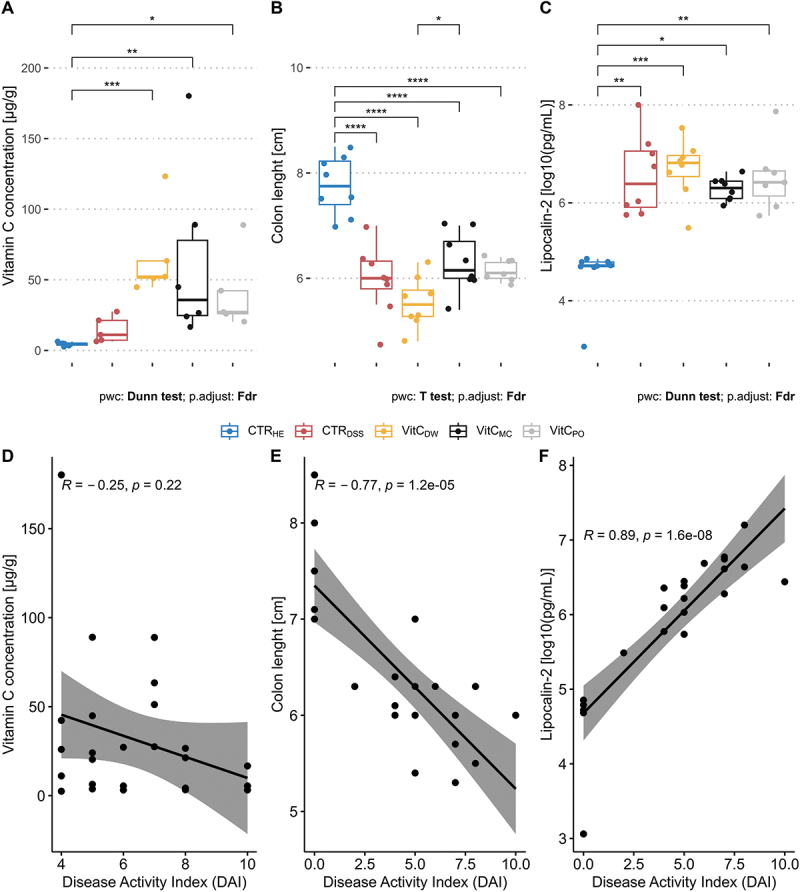
Vitamin C concentrations and disease markers on Day 7. A. Boxplot showing vitamin C concentrations [µg/g] in colon content. A non-parametric Kruskal Wallis test was performed, followed by a pairwise comparison using Dunn’s test with FDR adjustment. B. Boxplot showing colon length [cm]. A one-way ANOVA with FDR adjustment was performed within each day, followed by a pairwise *t* test with FDR adjustment between treatment groups for days with interactions found significant. C. Boxplot showing lipocalin-2 levels [log_10_(pg/mL)]. a non-parametric Kruskal Wallis test was performed, followed by a pairwise comparison using Dunn’s test with FDR adjustment. Individual measurements are represented by points in the boxplot. D. Scatterplot of disease activity index (DAI) against vitamin C concentrations [µg/g] in colon content. A non-parametric Spearman correlation test was performed, and a linear regression with 95% confidence interval. Healthy controls were excluded from the plot as their low DAI skewed the statistical test. Healthy controls (CTR_HE_, blue, A. *n* = 6, B-C. *n* = 8), DSS controls (CTR_DSS_, red, A. *n* = 5, B-C. *n* = 8), the drinking water group (VitC_DW_, yellow, A. *n* = 5, B-C. *n* = 8), the microcontainers group (VitC_MC_, black, A. *n* = 6, B-C. *n* = 8) and the powder group (VitC_PO_, grey, A. *n* = 4, B-C. *n* = 7). E. scatterplot of DAI against colon length [cm]. A non-parametric Spearman correlation test was performed, and a linear regression with 95% confidence interval. F. scatterplot of DAI against lipocalin-2 levels [log_10_(pg/mL)]. a non-parametric Spearman correlation test was performed, and a linear regression with 95% confidence interval. * = *p* < 0.05, ** = *p* < 0.01, *** = *p* < 0.001, **** = *p* < 0.0001.

Measurement of the vitamin C concentration in colon content revealed that vitamin C was present in comparable amounts in all three groups supplemented with vitamin C (Figure 3(A)). A significant difference to the untreated healthy controls (n = 6) was found, whereas the difference was not significant when comparing the drinking water group (Dunn’s test, n = 6, p.adj = 0.05), the microcontainers group (Dunn’s test, n = 6, p.adj = 0.2) and the powder group (Dunn’s test, n = 5, p.adj = 0.3) to DSS controls (n = 5). This shows that not only colon-targeted delivery by microcontainers, but also delivery as powder in peanut butter and in drinking water, led to presence of vitamin C in the colon.

Colon length comparisons indicated that worst disease symptoms were found in the drinking water group, with a significant shortening of the colon compared to the microcontainers group. However, significant shortenings were observed for all groups receiving DSS when compared to healthy controls (t-test, p.adj < 10^−5^, Figure 3(B), powder group, *n* = 7, other groups, *n* = 8). Levels of the inflammatory marker lipocalin-2 were likewise significantly higher for all groups receiving DSS compared to healthy controls (Figure 3(C), powder group, *n* = 7, other groups, *n* = 8).

We applied Spearman correlations to determine if the DAI were correlated with any of these three variables at day 7 (*n* = 24), independently of treatment groups (Figure 3(D–F)). DAI were negatively correlated with the colon length (spearman, p.adj < 10^−4^, Figure 3(E)) and positively correlated with the level of lipocalin-2 (spearman, p.adj < 10^−7^, Figure 3(F)).

On Day 7, gene expression of cytokines was measured in colon tissue. Reliable results for TNF-α were not obtained, and gene expression levels of IL-1β, IL6 and IL10 were below detection limit. The relative expression of IL-17 was significantly lower in healthy controls (*n* = 5) compared to the microcontainers group (*n* = 8) (Supplementary Figure S3). Despite similarities to the measurements of the vitamin C concentration in colon content (Figure 3(A)), no correlation to IL-17 expression was identified (*n* = 25, data not shown) and IL-17 did not correlate with DAI either (spearman, *n* = 21, p.adj = 0.11).

### Microbial dysbiosis and SCFAs

**Figure 4.**
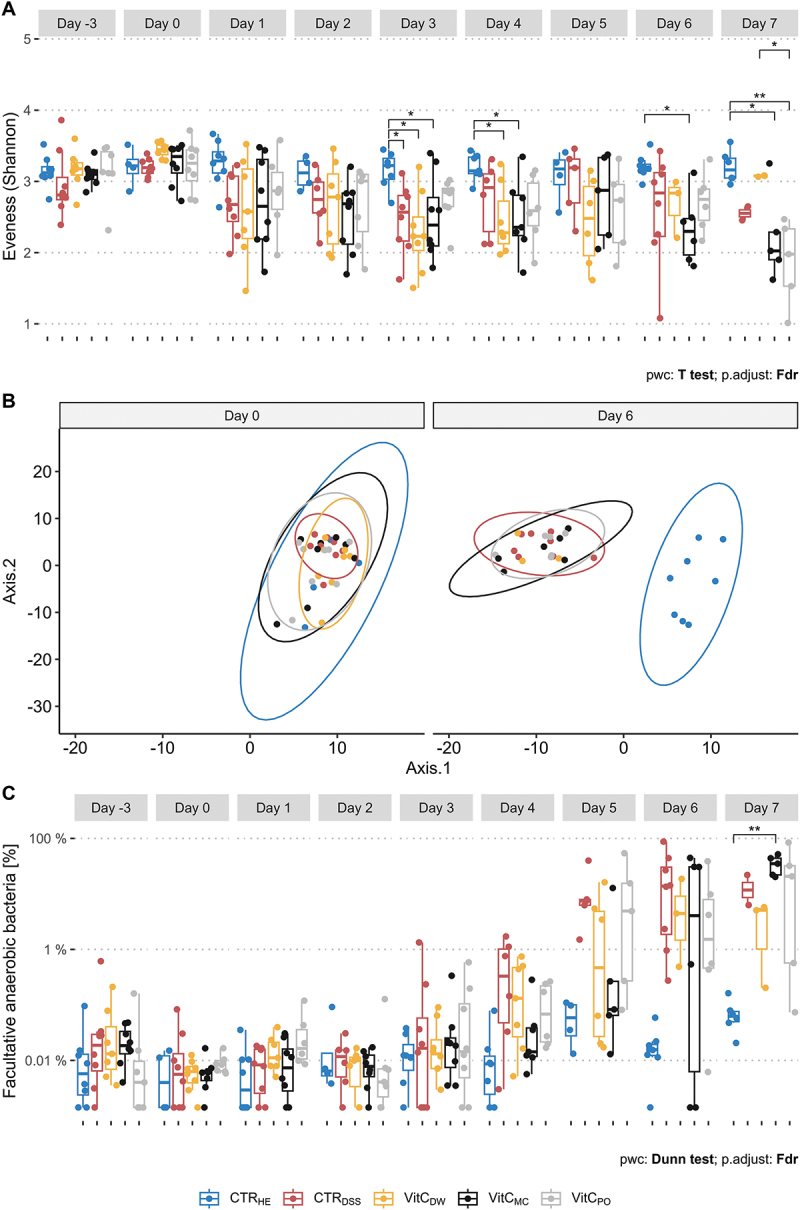
Microbial analysis. A. boxplot showing alpha diversity (evenness, Shannon diversity) on Days −3 and 0–7. The comparison was performed using a one-way ANOVA, followed by a pairwise *t* test with FDR adjustment. B. PCoA plot of beta diversity on Days 0 and 6. A PERMANOVA using the Aitchison distance matrix was applied. The beta dispersion for this matrix was found in-significant. C. levels of facultative anaerobic bacteria [%] on Days −3 and 0–7. Facultative anaerobic bacteria were here defined as the order *Enterobacterales*. A Kruskal Wallis test was performed, followed by a pairwise comparison using Dunn’s test with FDR adjustment. Healthy controls (CTR_HE_, blue), DSS controls (CTR_DSS_, red), the drinking water group (VitC_DW_, yellow), the microcontainers group (VitC_MC_, black) and the powder groups (VitC_PO_, grey). Measures for individual mice are included as points. * = *p* < 0.05, ** = *p* < 0.01, *** = *p* < 0.001.

Successful microbial analysis of 292 samples was performed (Supplementary Table S2), whereas 68 samples were excluded mainly due to lack of fecal samples and low DNA concentrations. Shannon diversity index (Shannon) (Figure 4(A)), Microbial richness (observed ASVs) (Supplementary Figure S4A) and phylogenetic diversity (FaithPD) (Supplementary Figure S4B) decreased in groups receiving DSS. This was most pronounced for the measures of richness and phylogenetic diversity, where significant decreases compared to healthy controls were observed from Day 1 and 2, respectively. At Day 3, an insignificant trend of higher richness in the microcontainers group (p.adj = 0.1) and the powder group (p.adj = 0.07) compared to the drinking water group was observed, whereas a significant opposite pattern was found at Day 7. Significant differences in beta diversity were found for healthy controls compared to the DSS-treated groups from Day 1–7 (Days 0 and 6: Figure 4(B), Days −3, 0–7: Supplementary Figure S5).

Feces of healthy mice were dominated by the phyla *Bacteroidetes* (genera: *Bacteroides*, *Phocaeicola*, and unknown within the order *Bacteroidales*) and *Firmicutes* (genera: *Intestinimonas*, unknown within the families *Lachnospiraceae* and *Ruminococcaceae*) (Supplementary Figure S6). All groups from Day −3 (before peanut butter adaption started) and Day 0 (before DSS-treatment started) were similar, showing that consumption of small amounts of peanut butter did not markedly affect the fecal microbiota within this setup. At Days 1–4, a shift to a high abundance of the genus *Phocaeicola* (phylum *Bacteroidetes*) was observed in all DSS-treated groups. However, at Days 5–7 bacteria of the facultative anaerobic genus *Escherichia/Shigella* (phylum *Proteobacteria*) took over, with a raise in the genus *Romboutsia* (phylum *Firmicutes*) as well.

In line with the above observations, differential abundance analysis for Days 3, 5 and 7 showed that healthy controls generally had significant higher abundances of various bacterial genera of the *Bacteroidetes* and *Firmicutes* phyla (Supplementary Table S3). At Day 7, an overall tendency of microbial recovery of the drinking water group was found, even though the observation is based on a small sample size (*n* = 3).

The estimated relative abundance of facultative anaerobic bacteria increased from Day 4 in all DSS-treated groups with large variations observed (Figure 4(C)). Vitamin C administration did not alleviate this dysbiosis.

For Days 5 and 7, SCFA levels were measured. Generally, lower levels of all SCFAs were observed on Day 7 compared to Day 5 (ending day for DSS). On Day 5, formic acid levels tended to be higher for all four groups administered DSS compared to healthy controls, with a significant finding for the powder group (Supplementary Figure S8). Measurements were mainly below detection level for 4-methyl-pentanoic acid (99% of the samples were below 0.09 µmol/g), hexanoic acid (92% of the samples were below 0.03 µmol/g) and heptanoic acid (all samples were below 0.03 µmol/g). For acetic acid, propanoic acid, 2-methyl-propanoic acid, butanoic acid, 3-methylbutanoic acid and pentanoic acid, no significant differences were observed between treatment groups (data not shown).

Comparing the bacterial genera at day 7 with the SCFA and clinical data, using a spearman correlation test, initially identified 125 significant correlations, out of 610, but only 3 passed adjustment for multiple testing; the genus *Paramuribaculum* was negatively correlated with IL-17 (rho = −0.84, p.adj < 10^−5^), while *Flavonifractor* was negatively correlated with acetic acid (rho = −0.82, p.adj < 10^−5^) and propanoic acid (rho = −0.79, p.adj < 10^−4^).

## Discussion

We hypothesized that colon-targeted delivery of vitamin C in microcontainers would alleviate disease markers and microbial dysbiosis in a colitis mouse model due to local release of the antioxidant and increased stabilization. We administered over three times more vitamin C in the drinking water group and due to assumed absorption in the small intestine we found statistically similar levels of vitamin C present in colon content within all groups dosed with vitamin C (Figure 3(A)). Hence, microcontainer delivery was not required to increase vitamin C levels in the colon. Despite similar colonic vitamin C concentrations, the three tested delivery strategies resulted in different disease development profiles. Delivery of vitamin C embedded in peanut butter, with or without prior incorporation in microcontainers, was observed to partially alleviate disease symptoms and delayed increase in DAI compared to DSS controls (Figure 2(D)). We suggest that both methods protect the active antioxidant form of vitamin C (AA: reduced form). In line with our observations, microencapsulation of AA in palm fat has previously been shown to markedly increase AA stability during storage.^18^ As the feces consistency and blood in the feces fluctuated in the individual mice, DAI should be interpreted as indicating a trend without a focus on the exact value, weight should be considered likewise.

Delivery of a high dose of vitamin C in the drinking water did not alleviate disease, since the symptoms were similar or worse than in DSS controls (Figure 2(C) and 3(B)). We suggest that this may be explained by the lack of stability of vitamin C in the drinking water. In prior studies, copper-dependent formation of hydrogen peroxide (H_2_O_2_) was observed in household drinking water supplemented with AA, through oxidation to DHAA.^38,39^ When delivered to the intestine, H_2_O_2_ can result in oxidative stress and tissue damage, supported by the observed association between increased levels of H_2_O_2_ and cell death in colon tissue.^14^ Furthermore, intracellular recycling of DHAA to AA may be impaired in subjects with increased oxidative stress states, including UC patients^12,40^ This suggests decreased antioxidant effects if DHAA is delivered to the diseased intestine. Measurements of reaction products of vitamin C in drinking water (e.g. H_2_O_2_) or levels of AA and DHAA separately, would be needed to verify these processes in future studies.

Analyses of the mouse fecal microbiome indicated that the dysbiosis associated with DSS-treatment was not alleviated by vitamin C administration. The microbial composition and SCFA measures did not reflect the worsened symptoms in the drinking water group, or the alleviating effects of vitamin C delivered in peanut butter (Figure 4A–C). Hence, the fecal microbiota was most likely not involved in the observed effects of vitamin C. In contrast to our results, prior studies suggest that the presence of vitamin C supports improved growth of strict anaerobic bacteria, production of SCFAs and increased alpha diversity^16,41^ However, our microbial results may be skewed by symptoms-related changes in behavior. The last days of study, several DSS-treated mice failed to deliver fecal samples and, in a few cases, to finish the peanut butter. This may introduce a bias by potentially not including the sickest animals in the analysis. Lastly, we dosed the mice with vitamin C for five days and this might be to short a supplementation period, at least too short for us to identify any shift in the microbiome. A longer time frame could potentially lead to more robust conclusions regarding the role of microbial dysbiosis

Gene expression of cytokines was expected to reflect pro-inflammatory immune responses in DSS treated mice. However, reliable detection of most investigated cytokines was not obtained. This could indicate that the DSS modeling was properly established. As the clinical symptoms did represent DSS well, it is our interpretation that the cytokine measurements were unreliable. Additional analysis of the cytokine expression would have been relevant to support the data presented in this study.

Our study emphasizes that the delivery strategy for vitamin C is very important. While vitamin C embedded in fatty matrices can partially alleviate colitis symptoms in DSS-treated mice, administration of this antioxidant in drinking water did not have a positive effect. Further investigations are needed to elucidate the relevance of these results in the context of oral delivery of antioxidants to human consumers, particularly UC patients.

## Supplementary Material

Supplementary material for vitamin C manuscript_revised.docx

## Data Availability

Scripts for complete data analysis are available at https://github.com/MSMortensen/VitC_delivery. and raw fastq files are uploaded to SRA, BioProject ID PRJNA1045461 (http://www.ncbi.nlm.nih.gov/bioproject/1045461).
