# Supplementary material for: Method of oral delivery affects vitamin C-mediated alleviation of colitis in a mouse model
Source: Gut Microbes Rep. 2025 Sep 18;2(1):2549734. doi: 10.1080/29933935.2025.2549734 (PMC12940098; doi:10.1080/29933935.2025.2549734)
Supplement: Supplementary material for vitamin C manuscript_revised.docx [file KGMR_A_2549734_SM7713.docx]

**Supplementary materials**

**Method of oral delivery affects vitamin C-mediated alleviation of colitis in a mouse model**

Pi Westi Bondegaard^1^, Katja Ann Kristensen^1^, Khorshid Kamguyan^2^, Jette Jakobsen^1^, Vanessa Emily Rees^3^, Mahdi Ghavami^2^, Line Hagner Nielsen^2^, Anja Boisen^2^, Martin Iain Bahl^1^, Tine Rask Licht^1^, Martin Steen Mortensen^1^

*^1^ National Food Institute, Technical University of Denmark, Kgs. Lyngby, 2800, Denmark
^2^ Department of Health Technology, Technical University of Denmark, Kgs. Lyngby, 2800, Denmark
^3^The Novo Nordisk Foundation Center for Biosustainability Technical University of Denmark, Kgs. Lyngby, 2800, Denmark*

**Correspondence to:** Martin Steen Mortensen, National Food Institute, Technical University of Denmark, Kgs. Lyngby, 2800, Kemitorvet, Building 202, Denmark. E-mail: masmo@food.dtu.dk; ORCID: 0000-0001-5483-7533

**Supplementary Figure 1.** Scanning electron microscope pictures of microcontainers on a chip after A) vitamin C loading and B) coating with Eudragit® FS100 and Eudragit® L100. The black scale bar represents 500 µm.

**A**


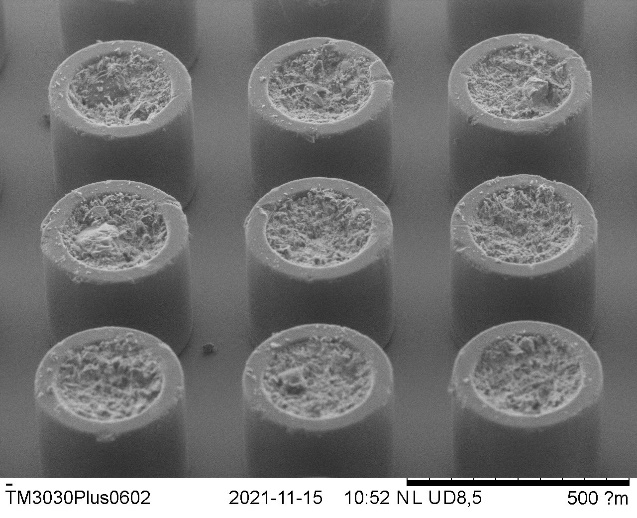

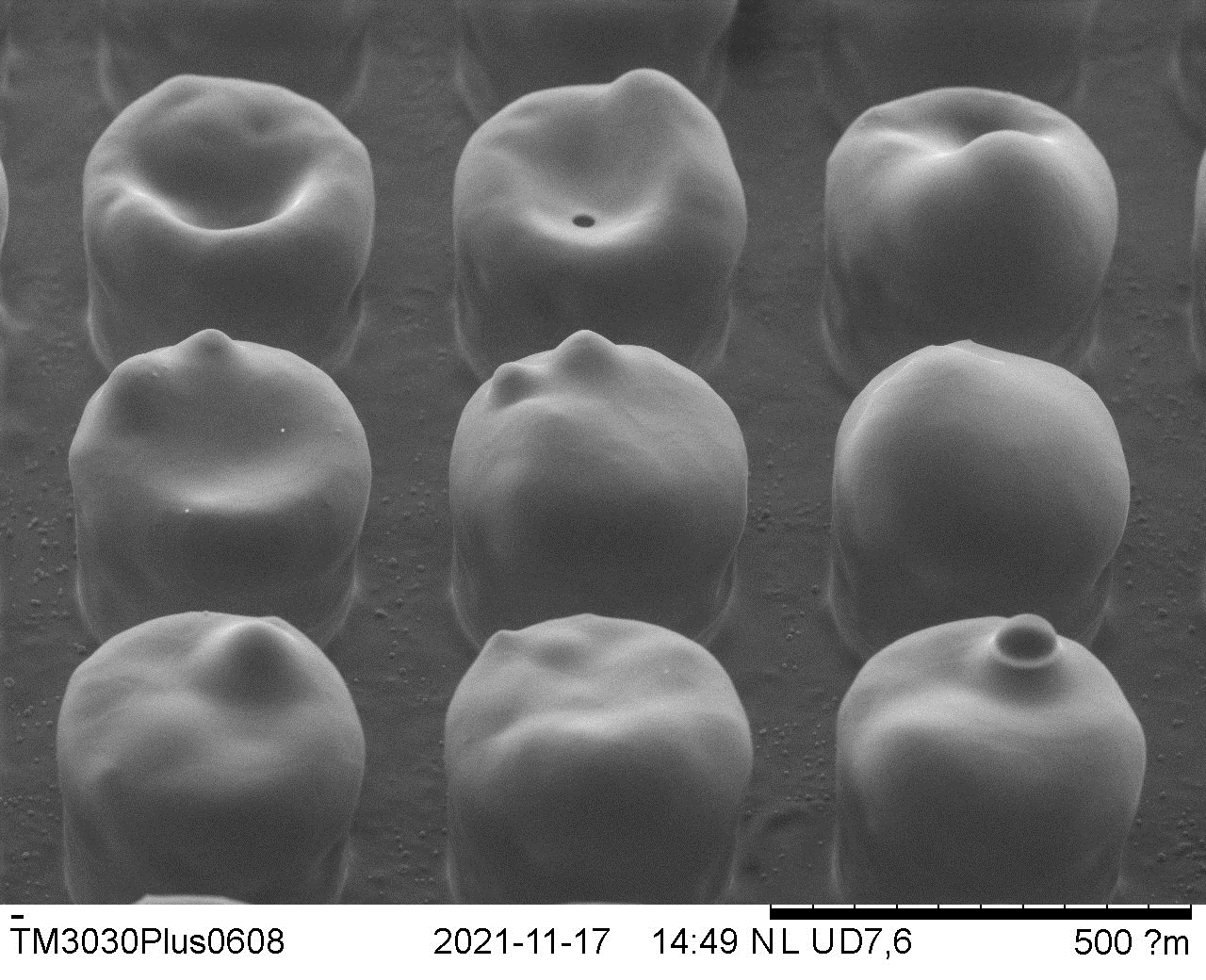


**B**


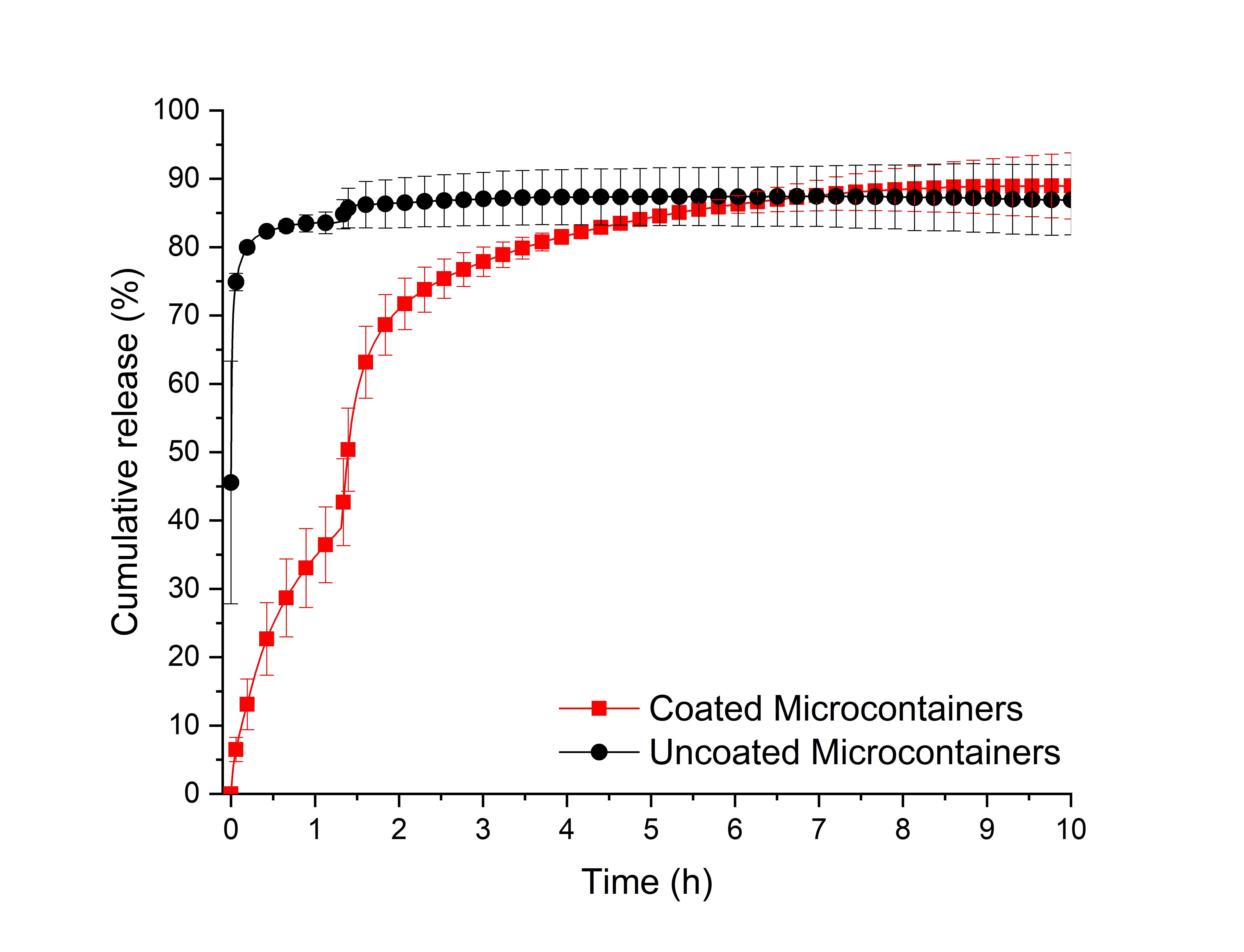


**Supplementary Figure 2.** *In vitro* release profile [%] for vitamin C from microcontainers coated with both Eudragit® FS100 and Eudragit® L100 (coated microcontainers, *n* = 3), or uncoated microcontainers (*n* = 3) in pH = 4.3 for 1 h followed by pH = 6.5 until end of experiment.


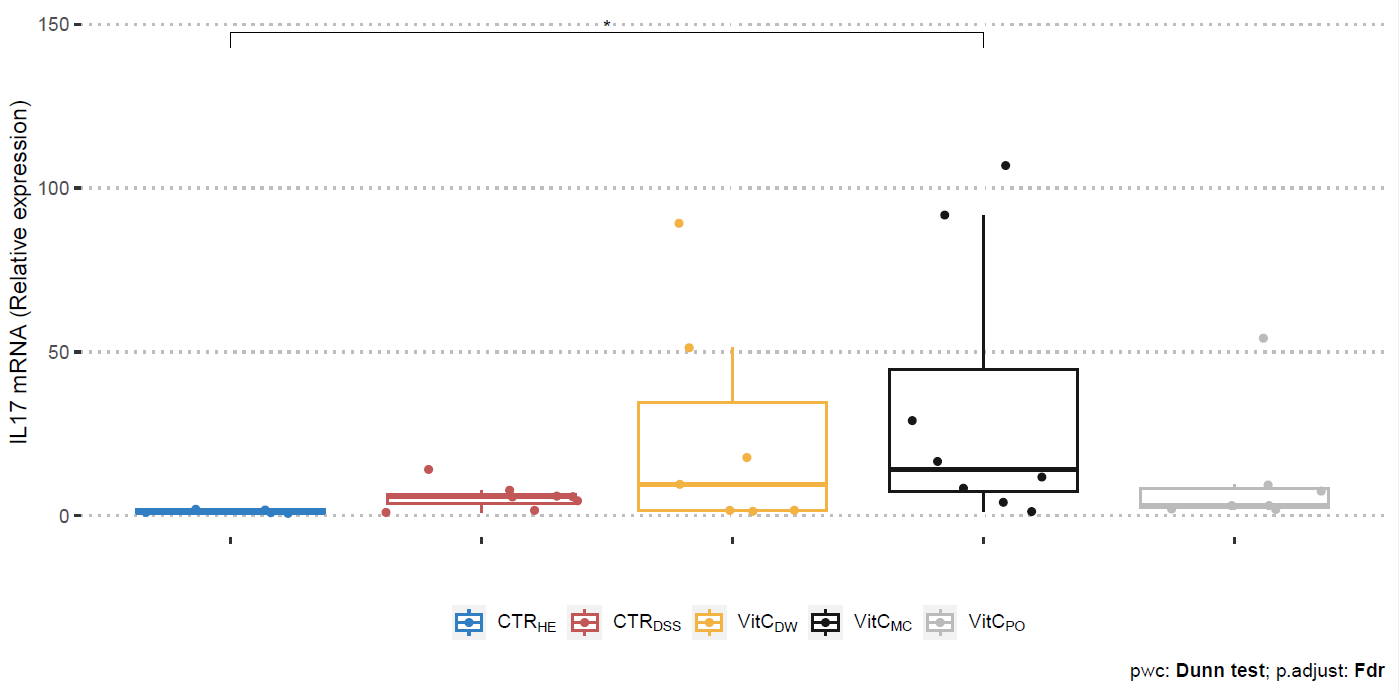


**Supplementary Figure 3.** Relative gene expression of IL-17 in colon tissue on Day 7. Healthy controls (CTR_HE_, blue, *n* = 5), DSS controls (CTR_DSS_, red, *n* = 8), the drinking water group (VitC_DW_, yellow, *n* = 7), the microcontainers group (VitC_MC_, black, *n* = 8) and the powder group (VitC_PO_, grey, *n* = 7). Individual measurements are represented by points in the boxplots. * = p < 0.05. Healthy controls vs. the microcontainers group (p = 0.011).


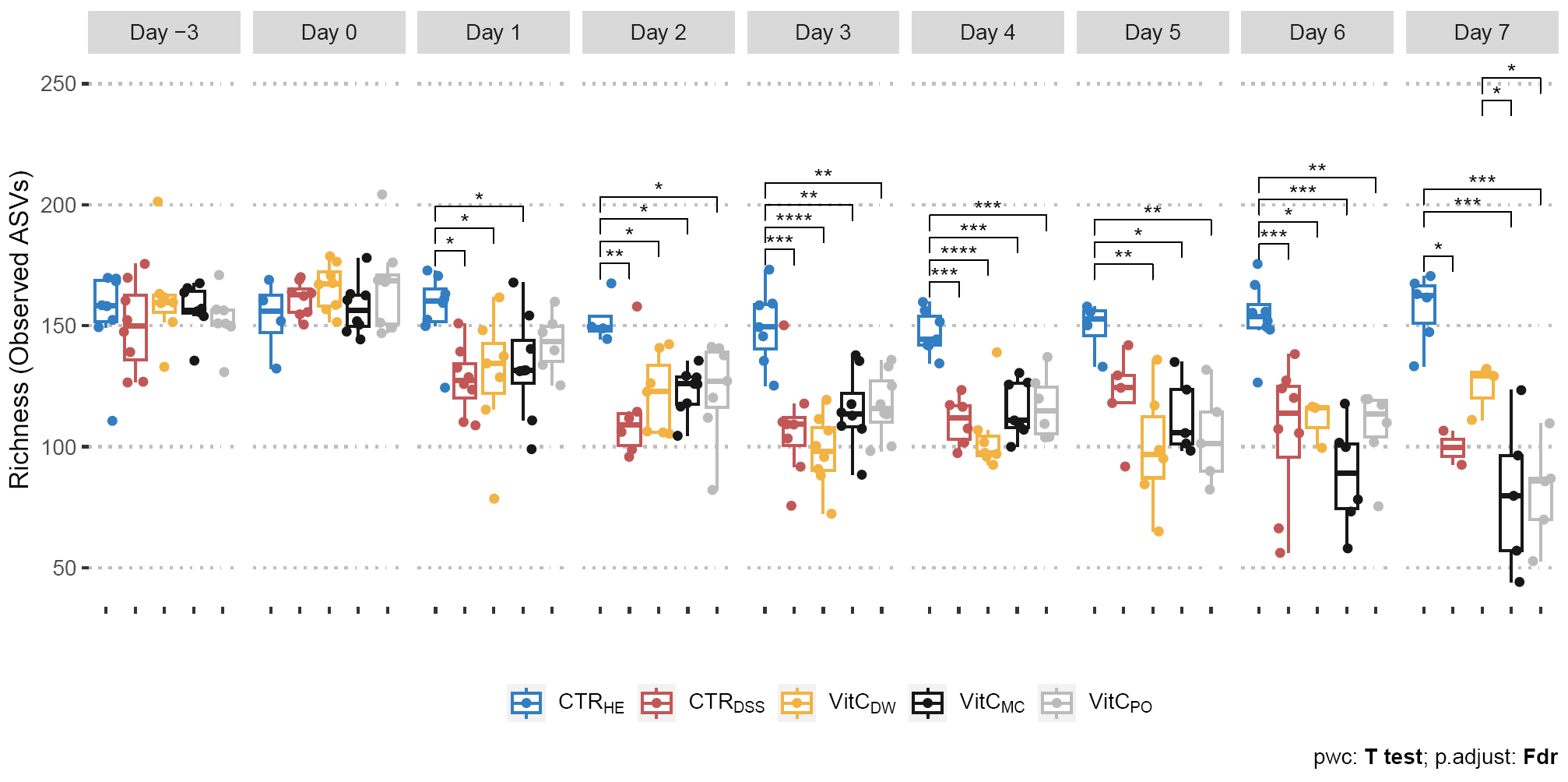


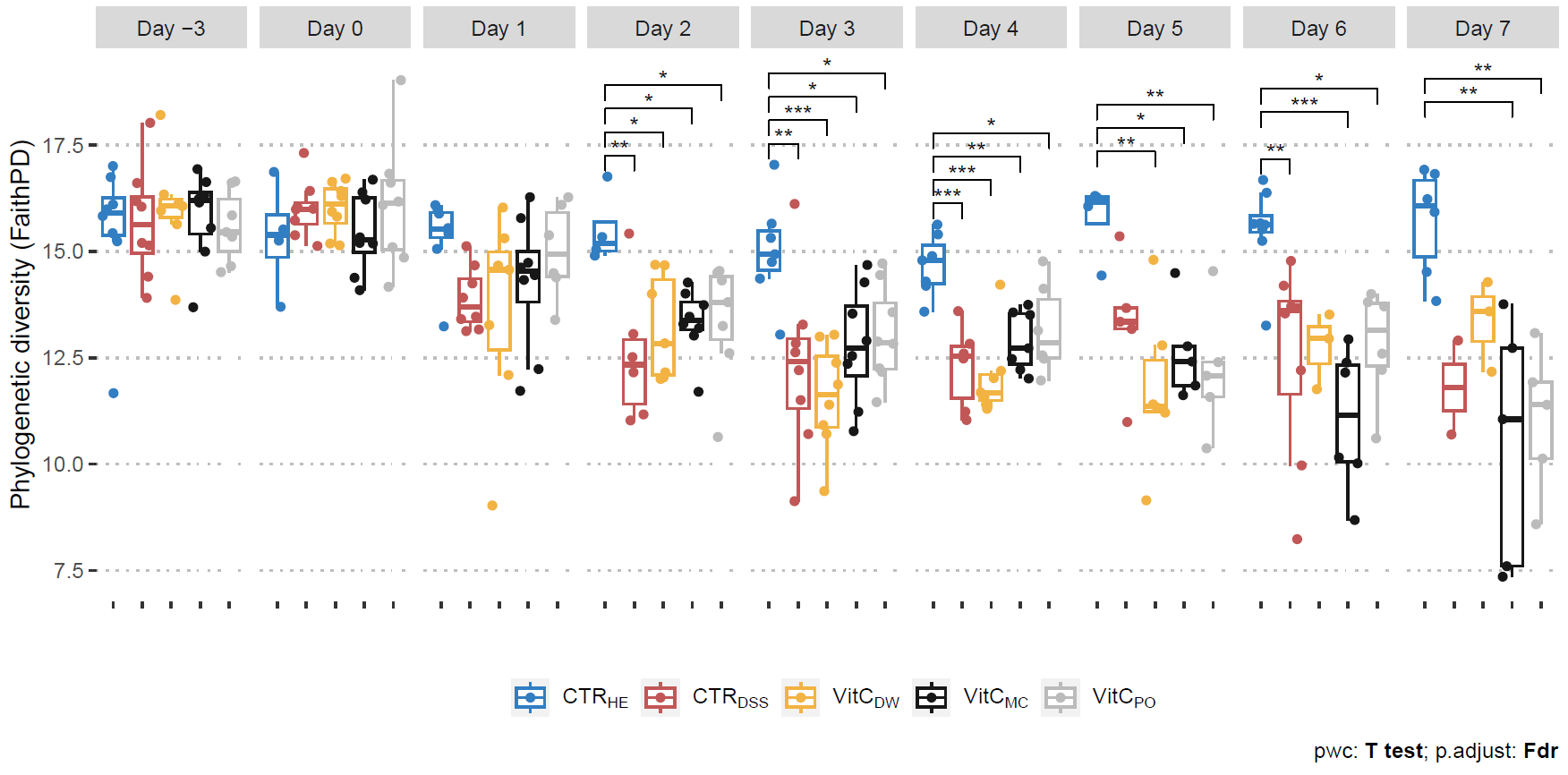


**Supplementary Figure 4A-B.** Alpha diversity. **A.** Richness (Observed ASVs), **B.** phylogenetic diversity (FaithPD). Healthy controls (CTR_HE_, blue), DSS controls (CTR_DSS_, red), the drinking water group (VitC_DW_, yellow), the microcontainers group (VitC_MC_, black) and the powder groups (VitC_PO_, grey). Measures for individual mice are included in the boxplots. * = p < 0.05, ** = p < 0.01, *** = p < 0.001.


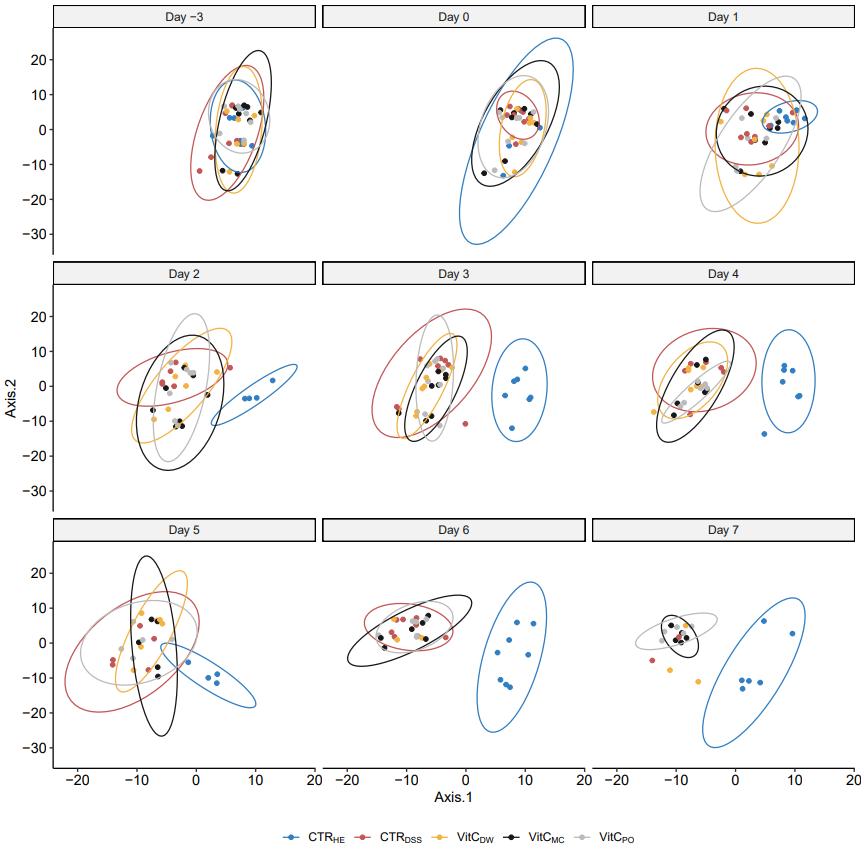


**Supplementary Figure 5.** PCoA plot of beta diversity using the Aitchison distance matrix on Day -3 and 0-7. Healthy controls (CTR_HE_, blue), DSS controls (CTR_DSS_, red), the drinking water group (VitC_DW_, yellow), the microcontainers group (VitC_MC_, black) and the powder groups (VitC_PO_, grey).


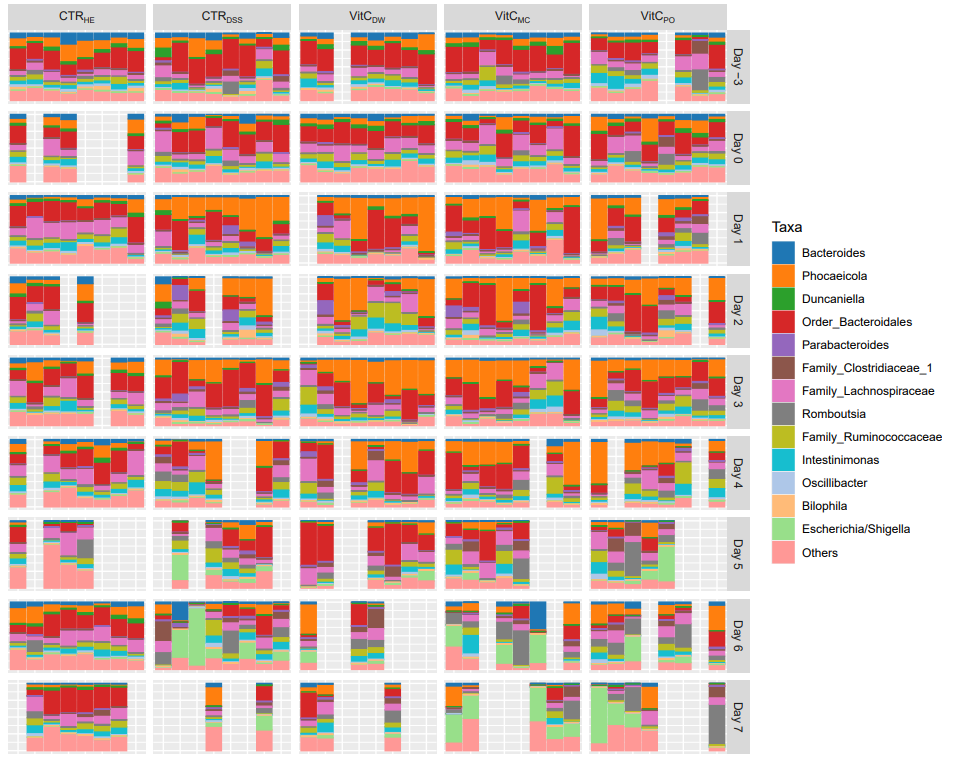


**Supplementary Figure 6.** Relative abundance (genus level) for Days -3 and 0-7. Individual animals are represented in each column.


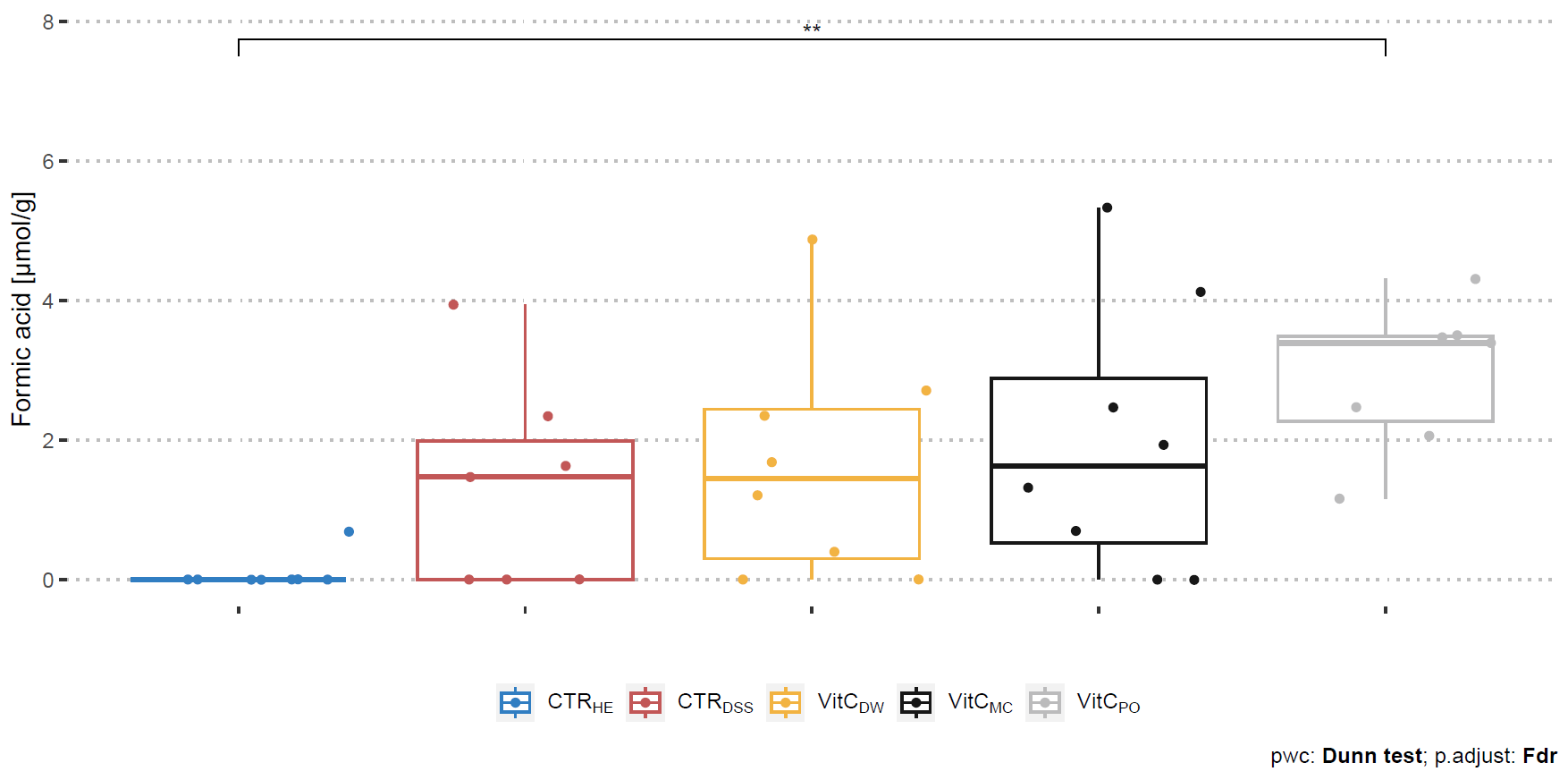


**Supplementary Figure 7.** Formic acid levels [µmol/g] on Day 5. Healthy controls (CTR_HE_, blue, *n* = 8), DSS controls (CTR_DSS_, red, *n* = 7), the drinking water group (VitC_DW_, yellow, *n* = 8), the microcontainers group (VitC_MC_, black, *n* = 8) and the powder groups (VitC_PO_, grey, *n* = 7). Individual measurements are represented by points. * = p < 0.05, ** = p < 0.01, *** = p < 0.001, **** = p < 0.0001. Healthy controls vs. the powder groups (p = 0.0025).

**Supplementary Table 1. List of primers used in the gene expression analysis of cytokines.**

| Endogenous TATA-box-binding protein (Tbp) gene | FWD 5’-ACCCTTCACCAATGACTCCTATG-3’  REV 5’-TGACTGCAGCAAATCGCTTGG-3’ |
| --- | --- |
| IL-17b | FWD 5’-GAGTAAAGCCCTACGCTCGAA-3’  REV 5’-CTCCTCTTGTTGGACAACCAC-3’ |
| TNF-α | FWD 5’-CATCCTTGCGAGTGTCAGTGA-3’  REV 5’-CCCTCACACTCAGATCATCTTCT-3’ |
| IL-1β | FWD 5’-GCAACTGTTCCTGAACTCAACT-3’  REV 5’-ATCTTTTGGGGTCCGTCAACT-3’ |
| IL-6 | FWD 5’-TGATGCACTTGCAGAAAACA-3’  REV 5’-GGTACTCCAGAAGACCAGAGGA-3’ |
| IL-10 | FWD 5’-AGTGGAGCAGGTGAAGAGTG-3’  REV 5’-CACTGCAGGTGTTTTAGCTTT-3’ |

**Supplementary Table 2. Number of microbiome samples per day and treatment**

| Treatment | Day -3 | Day 0 | Day 1 | Day 2 | Day 3 | Day 4 | Day 5 | Day 6 | Day 7 |
| --- | --- | --- | --- | --- | --- | --- | --- | --- | --- |
| DSS ctr | 8 | 8 | 8 | 6 | 8 | 6 | 5 | 8 | 2 |
| Healthy ctr | 8 | 4 | 8 | 4 | 7 | 7 | 4 | 8 | 6 |
| Vit C - Drinking water | 7 | 8 | 7 | 7 | 8 | 7 | 6 | 3 | 3 |
| Vit C - Microcontainers | 8 | 8 | 8 | 8 | 8 | 7 | 5 | 6 | 5 |
| Vit C - Powder | 7 | 8 | 6 | 7 | 8 | 6 | 5 | 6 | 5 |

**Supplementary Table 3. Significantly differential abundant genera at day 3, 5, and 7.**

| Day | Phylum | Genus | pval | pval.adj | prev_Healthy ctr | prev_DSS ctr | prev_Vit C - Drinking water | prev_Vit C - Microcontainers | prev_Vit C - Powder | mean_Healthy ctr | mean_DSS ctr | mean_Vit C - Drinking water | mean_Vit C - Microcontainers | mean_Vit C - Powder |
| --- | --- | --- | --- | --- | --- | --- | --- | --- | --- | --- | --- | --- | --- | --- |
| 3 | Bacteroidetes | Alistipes | 3.5E-07 | 2.7E-06 | 7 | 7 | 6 | 4 | 8 | 1.4104 | 0.1466 | 0.0949 | 0.0552 | 0.0566 |
| 3 | Firmicutes | Anaerotruncus | 2.3E-08 | 2.4E-07 | 7 | 8 | 8 | 8 | 8 | 0.8583 | 0.0529 | 0.1093 | 0.2945 | 0.0772 |
| 3 | Bacteroidetes | Bacteroides | 5.5E-08 | 4.9E-07 | 7 | 8 | 8 | 8 | 8 | 4.1077 | 1.3205 | 1.8091 | 1.3935 | 1.0946 |
| 3 | Firmicutes | Class Clostridia | 2.1E-02 | 4.8E-02 | 5 | 3 | 1 | 1 | 3 | 0.0113 | 0.0110 | 0.0006 | 0.0003 | 0.0066 |
| 3 | Firmicutes | Clostridium XlVa | 1.8E-05 | 1.1E-04 | 5 | 8 | 8 | 5 | 6 | 0.0034 | 0.0206 | 0.0143 | 0.0073 | 0.0172 |
| 3 | Firmicutes | Clostridium XVIII | 2.0E-02 | 4.6E-02 | 7 | 8 | 8 | 8 | 8 | 0.2938 | 0.0534 | 0.0978 | 0.0341 | 0.0317 |
| 3 | Firmicutes | Colidextribacter | 2.0E-02 | 4.6E-02 | 3 | 3 | 0 | 6 | 3 | 0.0339 | 0.0136 | - | 0.0128 | 0.0145 |
| 3 | Bacteroidetes | Duncaniella | 2.1E-24 | 1.3E-22 | 7 | 5 | 3 | 6 | 6 | 1.6139 | 0.1519 | 0.0031 | 0.0429 | 0.0263 |
| 3 | Firmicutes | Eubacterium | 2.8E-05 | 1.6E-04 | 7 | 6 | 3 | 5 | 3 | 1.0784 | 0.0025 | 0.0018 | 0.0843 | 0.0032 |
| 3 | Firmicutes | Faecalibaculum | 3.6E-05 | 1.8E-04 | 1 | 0 | 0 | 1 | 3 | 0.0003 | - | - | 0.0002 | 0.0113 |
| 3 | Firmicutes | Fam. Clostridiaceae 1 | 1.2E-03 | 4.5E-03 | 6 | 7 | 3 | 8 | 6 | 0.0314 | 1.1896 | 0.3804 | 0.1588 | 1.9907 |
| 3 | Bacteroidetes | Fam. Rikenellaceae | 4.6E-18 | 7.1E-17 | 7 | 3 | 2 | 4 | 5 | 0.8750 | 0.0919 | 0.0010 | 0.0143 | 0.0050 |
| 3 | Synergistetes | Fam. Synergistaceae | 3.0E-03 | 9.2E-03 | 7 | 2 | 4 | 8 | 7 | 0.3037 | 0.0211 | 0.0616 | 0.1522 | 0.0479 |
| 3 | Firmicutes | Ihubacter | 2.8E-04 | 1.1E-03 | 6 | 7 | 8 | 7 | 8 | 0.0786 | 0.1642 | 0.0269 | 0.0219 | 0.0281 |
| 3 | Firmicutes | Lawsonibacter | 2.5E-04 | 1.1E-03 | 7 | 8 | 6 | 6 | 7 | 0.0639 | 0.0165 | 0.0064 | 0.0183 | 0.0175 |
| 3 | Firmicutes | Ligilactobacillus | 2.0E-02 | 4.6E-02 | 7 | 8 | 8 | 8 | 8 | 0.5931 | 0.0722 | 0.1797 | 0.1255 | 0.2820 |
| 3 | Firmicutes | Limosilactobacillus | 1.6E-03 | 5.4E-03 | 2 | 1 | 2 | 3 | 3 | 0.0009 | 0.0003 | 0.0012 | 0.0012 | 0.0159 |
| 3 | Firmicutes | Neglecta | 7.6E-04 | 2.9E-03 | 7 | 7 | 8 | 8 | 8 | 0.0755 | 0.0204 | 0.0149 | 0.0409 | 0.0322 |
| 3 | Firmicutes | Order Clostridiales | 1.9E-09 | 2.3E-08 | 7 | 8 | 8 | 8 | 8 | 2.2128 | 0.4905 | 0.2249 | 0.3910 | 0.2975 |
| 3 | Bacteroidetes | Parabacteroides | 1.8E-04 | 8.7E-04 | 7 | 8 | 8 | 8 | 8 | 0.1563 | 4.5178 | 3.2025 | 2.3946 | 1.1448 |
| 3 | Firmicutes | Peptococcus | 2.1E-03 | 6.9E-03 | 0 | 0 | 0 | 2 | 1 | - | - | - | 0.3904 | 0.0004 |
| 3 | Bacteroidetes | Phocaeicola | 7.2E-03 | 2.0E-02 | 7 | 8 | 8 | 8 | 8 | 14.954 | 20.642 | 38.470 | 31.765 | 25.992 |
| 3 | Bacteroidetes | Phylum Bacteroidetes | 1.2E-22 | 3.7E-21 | 6 | 5 | 5 | 3 | 6 | 1.0810 | 0.0376 | 0.0171 | 0.0183 | 0.0267 |
| 3 | Firmicutes | Phylum Firmicutes | 4.1E-03 | 1.2E-02 | 7 | 8 | 8 | 8 | 8 | 0.5471 | 0.2529 | 0.2710 | 0.2755 | 0.2783 |
| 3 | Bacteroidetes | Prevotella | 3.3E-18 | 6.8E-17 | 5 | 2 | 0 | 0 | 0 | 0.0247 | 0.0005 | - | - | - |
| 3 | Firmicutes | Romboutsia | 8.3E-03 | 2.2E-02 | 7 | 8 | 7 | 8 | 8 | 0.3564 | 3.1682 | 0.6923 | 1.8979 | 6.3001 |
| 3 | Firmicutes | Staphylococcus | 1.9E-02 | 4.6E-02 | 5 | 3 | 3 | 4 | 2 | 0.0295 | 0.0042 | 0.0079 | 0.0024 | 0.0005 |
| 3 | Firmicutes | Streptococcus | 7.9E-06 | 5.4E-05 | 7 | 8 | 8 | 8 | 8 | 0.1054 | 0.0324 | 0.0217 | 0.0287 | 0.0277 |
| 5 | Firmicutes | Acutalibacter | 7.0E-04 | 1.9E-03 | 4 | 5 | 5 | 4 | 4 | 0.2059 | 0.0792 | 0.0171 | 0.0175 | 0.0385 |
| 5 | Firmicutes | Anaerofilum | 1.9E-04 | 5.5E-04 | 3 | 1 | 3 | 1 | 1 | 0.0226 | 0.0011 | 0.0027 | 0.0006 | 0.0008 |
| 5 | Firmicutes | Anaerotruncus | 3.8E-22 | 2.3E-20 | 4 | 5 | 6 | 4 | 4 | 1.7213 | 0.0975 | 0.0668 | 0.0682 | 0.0305 |
| 5 | Firmicutes | Class Clostridia | 4.0E-13 | 3.0E-12 | 3 | 1 | 5 | 1 | 0 | 0.1222 | 0.0025 | 0.0049 | 0.0044 | - |
| 5 | Firmicutes | Clostridium XlVa | 8.5E-19 | 1.7E-17 | 4 | 4 | 6 | 5 | 5 | 0.4832 | 0.0099 | 0.0376 | 0.0605 | 0.0390 |
| 5 | Firmicutes | Colidextribacter | 2.3E-18 | 3.5E-17 | 4 | 4 | 3 | 3 | 1 | 0.3479 | 0.0103 | 0.0103 | 0.0021 | 0.0005 |
| 5 | Bacteroidetes | Duncaniella | 4.4E-06 | 1.9E-05 | 4 | 5 | 5 | 3 | 2 | 0.5478 | 0.1366 | 0.0058 | 0.0135 | 0.0051 |
| 5 | Firmicutes | Enterococcus | 1.1E-05 | 4.0E-05 | 4 | 5 | 5 | 5 | 5 | 0.1244 | 3.5015 | 0.0674 | 0.8246 | 0.8216 |
| 5 | Firmicutes | Eubacterium | 2.9E-05 | 1.0E-04 | 3 | 3 | 5 | 3 | 4 | 3.5675 | 0.1743 | 0.0419 | 0.3852 | 0.0024 |
| 5 | Firmicutes | Faecalibaculum | 1.1E-02 | 2.8E-02 | 2 | 3 | 2 | 1 | 3 | 0.0013 | 0.0370 | 0.0223 | 0.0005 | 0.2125 |
| 5 | Actinobacteria | Fam. Eggerthellaceae | 9.0E-08 | 4.2E-07 | 4 | 5 | 6 | 4 | 5 | 0.1995 | 0.0440 | 0.0312 | 0.0214 | 0.0233 |
| 5 | Firmicutes | Fam. Peptostreptococcaceae | 1.3E-02 | 3.4E-02 | 1 | 2 | 0 | 1 | 2 | 0.0022 | 0.0042 | - | 0.0005 | 0.0120 |
| 5 | Bacteroidetes | Fam. Rikenellaceae | 2.7E-14 | 2.7E-13 | 4 | 4 | 2 | 2 | 1 | 0.1534 | 0.0032 | 0.0007 | 0.0123 | 0.0005 |
| 5 | Firmicutes | Flavonifractor | 8.0E-08 | 4.1E-07 | 4 | 4 | 5 | 5 | 5 | 0.5227 | 0.0616 | 0.0457 | 0.0883 | 0.0657 |
| 5 | Firmicutes | Lawsonibacter | 9.2E-05 | 3.0E-04 | 3 | 5 | 6 | 5 | 5 | 0.0999 | 0.0255 | 0.0171 | 0.0054 | 0.0115 |
| 5 | Firmicutes | Neglecta | 1.4E-09 | 8.0E-09 | 4 | 5 | 5 | 5 | 5 | 0.2646 | 0.0276 | 0.0237 | 0.0359 | 0.0329 |
| 5 | Bacteroidetes | Order Bacteroidales | 8.3E-05 | 2.8E-04 | 4 | 5 | 6 | 5 | 5 | 12.342 | 21.347 | 45.297 | 23.336 | 8.8110 |
| 5 | Firmicutes | Order Clostridiales | 8.7E-21 | 2.7E-19 | 4 | 5 | 6 | 5 | 5 | 21.815 | 0.6194 | 0.2687 | 0.2475 | 0.2055 |
| 5 | Firmicutes | Peptococcus | 3.8E-10 | 2.5E-09 | 0 | 0 | 0 | 2 | 0 | - | - | - | 0.0125 | - |
| 5 | Bacteroidetes | Phylum Bacteroidetes | 6.9E-06 | 2.8E-05 | 0 | 1 | 4 | 3 | 0 | - | 0.0017 | 0.0813 | 0.0366 | - |
| 5 | Firmicutes | Ruthenibacterium | 1.7E-17 | 2.0E-16 | 3 | 3 | 0 | 3 | 0 | 0.1980 | 0.0062 | - | 0.0026 | - |
| 5 | Firmicutes | Staphylococcus | 4.9E-10 | 3.0E-09 | 3 | 1 | 3 | 3 | 1 | 0.1283 | 0.0021 | 0.0035 | 0.0091 | 0.0011 |
| 5 | Firmicutes | Streptococcus | 3.4E-13 | 3.0E-12 | 4 | 5 | 6 | 5 | 5 | 0.8982 | 0.1129 | 0.0471 | 0.0719 | 0.0982 |
| 7 | Firmicutes | Acutalibacter | 2.7E-07 | 1.3E-06 | 6 | 1 | 2 | 3 | 1 | 0.1456 | 0.0034 | 0.0322 | 0.0107 | 0.0022 |
| 7 | Actinobacteria | Adlercreutzia | 1.8E-03 | 4.0E-03 | 5 | 1 | 2 | 2 | 4 | 0.1141 | 1.2506 | 0.8201 | 0.0662 | 1.4141 |
| 7 | Firmicutes | Agathobaculum | 2.7E-10 | 2.1E-09 | 6 | 0 | 2 | 1 | 2 | 0.0271 | - | 0.0015 | 0.0009 | 0.0017 |
| 7 | Firmicutes | Anaerostipes | 6.0E-04 | 1.5E-03 | 4 | 1 | 1 | 1 | 1 | 0.0356 | 0.0032 | 0.0016 | 0.0018 | 0.0011 |
| 7 | Firmicutes | Anaerotignum | 1.6E-02 | 2.9E-02 | 6 | 2 | 3 | 5 | 5 | 0.2014 | 0.0869 | 0.6265 | 0.5351 | 0.0975 |
| 7 | Firmicutes | Class Clostridia | 2.9E-12 | 2.6E-11 | 5 | 0 | 2 | 1 | 0 | 0.0600 | - | 0.0028 | 0.0006 | - |
| 7 | Firmicutes | Clostridium sensu stricto | 2.0E-04 | 5.8E-04 | 6 | 2 | 3 | 1 | 2 | 0.2747 | 0.0049 | 0.0490 | 0.0006 | 0.0074 |
| 7 | Firmicutes | Clostridium XlVa | 2.4E-24 | 4.9E-23 | 0 | 0 | 0 | 2 | 2 | - | - | - | 10.220 | 0.0287 |
| 7 | Firmicutes | Colidextribacter | 4.0E-19 | 6.2E-18 | 5 | 0 | 2 | 0 | 2 | 0.2535 | - | 0.0098 | - | 0.0084 |
| 7 | Bacteroidetes | Duncaniella | 9.6E-43 | 5.9E-41 | 6 | 2 | 3 | 3 | 1 | 2.4206 | 0.0368 | 0.0024 | 0.0229 | 0.0040 |
| 7 | Firmicutes | Enterococcus | 1.3E-07 | 6.8E-07 | 5 | 2 | 3 | 5 | 5 | 0.0543 | 14.154 | 1.0963 | 9.8672 | 8.2088 |
| 7 | Proteobacteria | Escherichia/Shigella | 3.7E-06 | 1.7E-05 | 6 | 2 | 3 | 5 | 5 | 0.0711 | 14.110 | 3.6756 | 34.621 | 27.606 |
| 7 | Proteobacteria | Fam. Desulfovibrionaceae | 2.7E-03 | 5.8E-03 | 6 | 2 | 3 | 5 | 5 | 4.3826 | 1.0294 | 4.2688 | 1.5206 | 0.3044 |
| 7 | Actinobacteria | Fam. Eggerthellaceae | 4.6E-04 | 1.2E-03 | 6 | 2 | 3 | 2 | 4 | 0.1338 | 0.0242 | 0.0793 | 0.0063 | 0.0163 |
| 7 | Firmicutes | Fam. Lachnospiraceae | 1.0E-02 | 2.0E-02 | 6 | 2 | 3 | 5 | 5 | 15.592 | 5.3389 | 13.200 | 3.5187 | 7.7480 |
| 7 | Bacteroidetes | Fam. Muribaculaceae | 1.1E-05 | 4.7E-05 | 6 | 2 | 3 | 2 | 5 | 1.7081 | 0.2684 | 2.0387 | 0.1161 | 0.3479 |
| 7 | Firmicutes | Fam. Peptostreptococcaceae | 5.2E-04 | 1.4E-03 | 2 | 1 | 1 | 0 | 1 | 0.0007 | 0.0145 | 0.0022 | - | 0.0046 |
| 7 | Bacteroidetes | Fam. Rikenellaceae | 1.2E-08 | 7.5E-08 | 6 | 0 | 0 | 2 | 1 | 0.1676 | - | - | 0.0303 | 0.0057 |
| 7 | Firmicutes | Fam. Ruminococcaceae | 1.7E-04 | 5.1E-04 | 6 | 2 | 3 | 5 | 5 | 8.3328 | 1.7914 | 6.7250 | 2.8049 | 0.7578 |
| 7 | Synergistetes | Fam. Synergistaceae | 5.0E-05 | 1.6E-04 | 6 | 2 | 2 | 2 | 3 | 0.4451 | 0.0228 | 0.1354 | 0.0290 | 0.0134 |
| 7 | Firmicutes | Flavonifractor | 1.0E-30 | 3.1E-29 | 6 | 1 | 3 | 3 | 2 | 0.1818 | 0.0095 | 0.0143 | 0.0059 | 0.0045 |
| 7 | Firmicutes | Intestinimonas | 1.6E-03 | 3.6E-03 | 6 | 2 | 3 | 5 | 5 | 7.6710 | 2.4956 | 9.4229 | 2.6401 | 1.2653 |
| 7 | Firmicutes | Lawsonibacter | 4.7E-08 | 2.6E-07 | 6 | 2 | 3 | 3 | 2 | 0.1289 | 0.0215 | 0.0322 | 0.0082 | 0.0015 |
| 7 | Deferribacteres | Mucispirillum | 8.8E-03 | 1.8E-02 | 5 | 0 | 3 | 3 | 2 | 0.0614 | - | 0.1135 | 0.0298 | 0.0047 |
| 7 | Firmicutes | Neglecta | 4.5E-14 | 5.6E-13 | 6 | 2 | 2 | 2 | 4 | 0.1638 | 0.0049 | 0.0707 | 0.0081 | 0.0046 |
| 7 | Bacteroidetes | Order Bacteroidales | 1.6E-05 | 6.1E-05 | 6 | 2 | 3 | 5 | 5 | 30.998 | 11.808 | 18.827 | 4.4098 | 2.3034 |
| 7 | Firmicutes | Order Clostridiales | 2.4E-13 | 2.5E-12 | 6 | 2 | 3 | 3 | 5 | 8.8493 | 0.0967 | 0.3520 | 0.0417 | 0.1766 |
| 7 | Firmicutes | Oscillibacter | 2.1E-05 | 7.4E-05 | 6 | 2 | 3 | 4 | 5 | 2.2789 | 0.3031 | 1.2482 | 0.3313 | 0.1452 |
| 7 | Bacteroidetes | Parabacteroides | 1.5E-03 | 3.5E-03 | 6 | 2 | 3 | 5 | 5 | 0.0517 | 3.2618 | 1.4525 | 0.7868 | 0.1841 |
| 7 | Bacteroidetes | Paramuribaculum | 2.1E-05 | 7.4E-05 | 6 | 2 | 3 | 4 | 5 | 0.8716 | 0.0796 | 1.0445 | 0.0539 | 0.0654 |
| 7 | Bacteroidetes | Phylum Bacteroidetes | 2.2E-02 | 4.0E-02 | 2 | 0 | 0 | 0 | 3 | 0.1297 | - | - | - | 0.0034 |
| 7 | Firmicutes | Phylum Firmicutes | 1.6E-02 | 2.9E-02 | 6 | 2 | 3 | 4 | 5 | 0.8122 | 2.6776 | 0.0392 | 0.0683 | 0.1602 |
| 7 | Bacteroidetes | Prevotella | 4.6E-09 | 3.2E-08 | 3 | 0 | 0 | 0 | 0 | 0.0146 | - | - | - | - |
| 7 | Firmicutes | Ruthenibacterium | 4.7E-05 | 1.5E-04 | 4 | 1 | 3 | 2 | 1 | 0.1134 | 0.0009 | 0.0056 | 0.0015 | 0.0004 |
| 7 | Proteobacteria | Turicimonas | 9.5E-03 | 1.9E-02 | 1 | 1 | 2 | 3 | 2 | 0.0003 | 0.0009 | 0.0510 | 0.2993 | 0.1521 |

**Supplementary statistical test results**

**Figure 2A.**
Within each day:
Day 2: CTR_HE_ vs. VitC_DW_ (p = 0.015), VitC_DW_ vs. VitC_MC_ (p = 0.043), VitC_DW_ vs. VitC_PO_ (p = 0.045).
Day 3: CTR_HE_ vs. CTR_DSS_ (p = 0.033), CTR_HE_ vs. VitC_DW_ (p = 0.023).
Day 4: CTR_HE_ vs. CTR_DSS_ (p = 0.0042), CTR_HE_ vs. VitC_DW_ (p = 0.023), CTR_DSS_ vs. VitC_PO_ (p = 0.032).
Day 5: CTR_HE_ vs. CTR_DSS_ (p = 0.0018), CTR_HE_ vs. VitC_DW_ (p = 0.015), CTR_DSS_ vs. VitC_PO_ (p = 0.032).
Day 6: CTR_HE_ vs. CTR_DSS_ (p = 0.0013), CTR_HE_ vs. VitC_DW_ (p = 0.0042), CTR_DSS_ vs. VitC_PO_ (p = 0.028).
Day 7: CTR_HE_ vs. CTR_DSS_ (p = 0.00021), CTR_HE_ vs. VitC_DW_ (p = 0.00026), CTR_HE_ vs. VitC_MC_ (p = 0.015), CTR_HE_ vs. VitC_PO_ (p = 0.043), CTR_DSS_ vs. VitC_PO_ (p = 0.038).

**Figure 3A-C.**
**A.** CTR_HE_ vs. VitC_DW_ (p = 0.0010), CTR_HE_ vs. VitC_MC_ (p = 0.0060), CTR_HE_ vs. VitC_PO_ (p = 0.021).
**B.** CTR_HE_ vs. CTR_DSS_ (p = 0.00000041), CTR_HE_ vs. VitC_DW_ (p = 0.0000000071), CTR_HE_ vs. VitC_MC_ (p = 0.0000070), CTR_HE_ vs. VitC_PO_ (p = 0.0000034), VitC_DW_ vs. VitC_MC_ (p = 0.015).
**C.** CTR_HE_ vs. CTR_DSS_ (p = 0.0029), CTR_HE_ vs. VitC_DW_ (p = 0.00037), CTR_HE_ vs. VitC_MC_ (p = 0.010), CTR_HE_ vs. VitC_PO_ (p = 0.0062).

**Figure 4A.**

| Day | group1 | group2 | n1 | n2 | p | p.signif | p.adj | p.adj.signif |
| --- | --- | --- | --- | --- | --- | --- | --- | --- |
| Day 1 | CTR_HE_ | CTR_DSS_ | 8 | 8 | 4.11E-03 | ** | 0.016022 | * |
| Day 1 | CTR_HE_ | VitC_DW_ | 8 | 7 | 8.25E-03 | ** | 0.026441 | * |
| Day 1 | CTR_HE_ | VitC_MC_ | 8 | 8 | 1.87E-02 | * | 0.048481 | * |
| Day 2 | CTR_HE_ | CTR_DSS_ | 4 | 6 | 1.57E-03 | ** | 0.007327 | ** |
| Day 2 | CTR_HE_ | VitC_DW_ | 4 | 7 | 6.68E-03 | ** | 0.02338 | * |
| Day 2 | CTR_HE_ | VitC_MC_ | 4 | 8 | 8.31E-03 | ** | 0.026441 | * |
| Day 2 | CTR_HE_ | VitC_PO_ | 4 | 7 | 9.68E-03 | ** | 0.029461 | * |
| Day 3 | CTR_HE_ | CTR_DSS_ | 7 | 8 | 3.33E-05 | **** | 0.000333 | *** |
| Day 3 | CTR_HE_ | VitC_DW_ | 7 | 8 | 9.16E-07 | **** | 3.21E-05 | **** |
| Day 3 | CTR_HE_ | VitC_MC_ | 7 | 8 | 3.42E-04 | *** | 0.002176 | ** |
| Day 3 | CTR_HE_ | VitC_PO_ | 7 | 8 | 6.40E-04 | *** | 0.003425 | ** |
| Day 4 | CTR_HE_ | CTR_DSS_ | 7 | 6 | 1.10E-05 | **** | 0.000193 | *** |
| Day 4 | CTR_HE_ | VitC_DW_ | 7 | 7 | 4.71E-07 | **** | 3.21E-05 | **** |
| Day 4 | CTR_HE_ | VitC_MC_ | 7 | 7 | 4.81E-05 | **** | 0.000421 | *** |
| Day 4 | CTR_HE_ | VitC_PO_ | 7 | 6 | 1.27E-04 | *** | 0.000889 | *** |
| Day 5 | CTR_HE_ | VitC_DW_ | 4 | 6 | 6.85E-04 | *** | 0.003425 | ** |
| Day 5 | CTR_HE_ | VitC_MC_ | 4 | 5 | 1.05E-02 | * | 0.030625 | * |
| Day 5 | CTR_HE_ | VitC_PO_ | 4 | 5 | 2.22E-03 | ** | 0.009713 | ** |
| Day 6 | CTR_HE_ | CTR_DSS_ | 8 | 8 | 1.10E-04 | *** | 0.000856 | *** |
| Day 6 | CTR_HE_ | VitC_DW_ | 8 | 3 | 5.80E-03 | ** | 0.021368 | * |
| Day 6 | CTR_HE_ | VitC_MC_ | 8 | 6 | 4.71E-06 | **** | 0.00011 | *** |
| Day 6 | CTR_HE_ | VitC_PO_ | 8 | 6 | 3.91E-04 | *** | 0.002281 | ** |
| Day 7 | CTR_HE_ | CTR_DSS_ | 6 | 2 | 4.12E-03 | ** | 0.016022 | * |
| Day 7 | CTR_HE_ | VitC_MC_ | 6 | 5 | 1.75E-05 | **** | 0.000231 | *** |
| Day 7 | VitC_DW_ | VitC_MC_ | 3 | 5 | 1.15E-02 | * | 0.0322 | * |
| Day 7 | CTR_HE_ | VitC_PO_ | 6 | 5 | 1.98E-05 | **** | 0.000231 | *** |
| Day 7 | VitC_DW_ | VitC_PO_ | 3 | 5 | 1.28E-02 | * | 0.034462 | * |

**Figure 4B and Supplementary Figure 5.**

**Day -3 and 0**: No significant differences

**Day 1:**

$`VitC_DW__vs_CTR_HE_`

Df SumOfSqs R2 F Pr(>F)

treatment 1 977.8 0.14884 2.2733 0.001 ***

Residual 13 5591.6 0.85116

Total 14 6569.4 1.00000

$`VitC_MC__vs_CTR_HE_`

Df SumOfSqs R2 F Pr(>F)

treatment 1 617.2 0.09511 1.4716 0.028 *

Residual 14 5872.0 0.90489

Total 15 6489.2 1.00000

$`VitC_PO__vs_CTR_DSS_`

Df SumOfSqs R2 F Pr(>F)

treatment 1 668.9 0.11565 1.5694 0.004 **

Residual 12 5114.5 0.88435

Total 13 5783.4 1.00000

$`VitC_PO__vs_CTR_HE_`

Df SumOfSqs R2 F Pr(>F)

treatment 1 922.9 0.15444 2.1918 0.001 ***

Residual 12 5052.9 0.84556

Total 13 5975.8 1.00000

$`CTR_DSS__vs_CTR_HE_`

Df SumOfSqs R2 F Pr(>F)

treatment 1 815.7 0.12959 2.0844 0.004 **

Residual 14 5478.9 0.87041

Total 15 6294.6 1.00000

**Day 2:**

$`VitC_DW__vs_CTR_HE_`

Df SumOfSqs R2 F Pr(>F)

treatment 1 995.3 0.21654 2.4875 0.003 **

Residual 9 3601.0 0.78346

Total 10 4596.3 1.00000

$`VitC_MC__vs_CTR_HE_`

Df SumOfSqs R2 F Pr(>F)

treatment 1 1010.3 0.2041 2.5644 0.003 **

Residual 10 3939.6 0.7959

Total 11 4949.9 1.0000

$`VitC_MC__vs_CTR_DSS_`

Df SumOfSqs R2 F Pr(>F)

treatment 1 560.5 0.10553 1.4157 0.027 *

Residual 12 4751.0 0.89447

Total 13 5311.5 1.00000

$`VitC_PO__vs_CTR_HE_`

Df SumOfSqs R2 F Pr(>F)

treatment 1 946.2 0.20613 2.3369 0.004 **

Residual 9 3644.1 0.79387

Total 10 4590.3 1.00000

$`CTR_HE__vs_CTR_DSS_`

Df SumOfSqs R2 F Pr(>F)

treatment 1 927.6 0.23081 2.4005 0.01 **

Residual 8 3091.3 0.76919

Total 9 4018.9 1.00000

**Day 3:**

$`CTR_HE__vs_CTR_DSS_`

Df SumOfSqs R2 F Pr(>F)

treatment 1 1361.8 0.19774 3.2041 0.001 ***

Residual 13 5525.2 0.80226

Total 14 6887.1 1.00000

$`CTR_HE__vs_VitC_DW_`

Df SumOfSqs R2 F Pr(>F)

treatment 1 1401.7 0.21425 3.5448 0.001 ***

Residual 13 5140.6 0.78575

Total 14 6542.4 1.00000

$`CTR_HE__vs_VitC_MC_`

Df SumOfSqs R2 F Pr(>F)

treatment 1 1284.7 0.19268 3.1027 0.001 ***

Residual 13 5382.6 0.80732

Total 14 6667.3 1.00000

$`CTR_HE__vs_VitC_PO_`

Df SumOfSqs R2 F Pr(>F)

treatment 1 1227.3 0.18797 3.0092 0.001 ***

Residual 13 5302.2 0.81203

Total 14 6529.5 1.00000

**Day 4:**

$`CTR_HE__vs_CTR_DSS_`

Df SumOfSqs R2 F Pr(>F)

treatment 1 1145.6 0.20005 2.7508 0.001 ***

Residual 11 4581.1 0.79995

Total 12 5726.8 1.00000

$`CTR_HE__vs_VitC_DW_`

Df SumOfSqs R2 F Pr(>F)

treatment 1 1418.8 0.22889 3.5619 0.001 ***

Residual 12 4780.0 0.77111

Total 13 6198.8 1.00000

$`CTR_HE__vs_VitC_MC_`

Df SumOfSqs R2 F Pr(>F)

treatment 1 1294.2 0.20868 3.1644 0.001 ***

Residual 12 4907.7 0.79132

Total 13 6201.8 1.00000

$`CTR_HE__vs_VitC_PO_`

Df SumOfSqs R2 F Pr(>F)

treatment 1 1189.6 0.22494 3.1925 0.002 **

Residual 11 4098.9 0.77506

Total 12 5288.5 1.00000

**Day 5:**

$`CTR_HE__vs_CTR_DSS_`

Df SumOfSqs R2 F Pr(>F)

treatment 1 1188.1 0.28389 2.775 0.009 **

Residual 7 2997.1 0.71611

Total 8 4185.2 1.00000

$`CTR_HE__vs_VitC_DW_`

Df SumOfSqs R2 F Pr(>F)

treatment 1 1293.6 0.27758 3.0739 0.006 **

Residual 8 3366.6 0.72242

Total 9 4660.2 1.00000

$`CTR_HE__vs_VitC_MC_`

Df SumOfSqs R2 F Pr(>F)

treatment 1 1065.3 0.27478 2.6523 0.012 *

Residual 7 2811.5 0.72522

Total 8 3876.7 1.00000

$`CTR_HE__vs_VitC_PO_`

Df SumOfSqs R2 F Pr(>F)

treatment 1 1138.5 0.29036 2.8642 0.008 **

Residual 7 2782.6 0.70964

Total 8 3921.1 1.00000

**Day 6:**

$`CTR_HE__vs_CTR_DSS_`

Df SumOfSqs R2 F Pr(>F)

treatment 1 1843.9 0.23154 4.2184 0.001 ***

Residual 14 6119.4 0.76846

Total 15 7963.3 1.00000

$`CTR_HE__vs_VitC_MC_`

Df SumOfSqs R2 F Pr(>F)

treatment 1 1499.0 0.23072 3.599 0.001 ***

Residual 12 4998.1 0.76928

Total 13 6497.2 1.00000

$`CTR_HE__vs_VitC_PO_`

Df SumOfSqs R2 F Pr(>F)

treatment 1 1557.0 0.23378 3.6614 0.001 ***

Residual 12 5102.8 0.76622

Total 13 6659.8 1.00000

$`CTR_HE__vs_VitC_DW_`

Df SumOfSqs R2 F Pr(>F)

treatment 1 1110.5 0.22695 2.6422 0.006 **

Residual 9 3782.8 0.77305

Total 10 4893.3 1.00000

**Day 7:**

$`CTR_HE__vs_CTR_DSS_`

Df SumOfSqs R2 F Pr(>F)

treatment 1 948.0 0.23712 1.865 0.045 *

Residual 6 3049.9 0.76288

Total 7 3997.9 1.00000

$`CTR_HE__vs_VitC_DW_`

Df SumOfSqs R2 F Pr(>F)

treatment 1 924.9 0.20567 1.8125 0.012 *

Residual 7 3572.1 0.79433

Total 8 4497.0 1.00000

$`CTR_HE__vs_VitC_PO_`

Df SumOfSqs R2 F Pr(>F)

treatment 1 1493.8 0.28206 3.5359 0.004 **

Residual 9 3802.1 0.71794

Total 10 5295.9 1.00000

$`CTR_HE__vs_VitC_MC_`

Df SumOfSqs R2 F Pr(>F)

treatment 1 1468.3 0.26567 3.2561 0.003 **

Residual 9 4058.5 0.73433

Total 10 5526.8 1.00000

**Supplementary Figure 4A.**

| Day | group1 | group2 | n1 | n2 | p | p.signif | p.adj | p.adj.signif |
| --- | --- | --- | --- | --- | --- | --- | --- | --- |
| Day 3 | CTR_HE_ | CTR_DSS_ | 7 | 8 | 0.00745 | ** | 0.0407 | * |
| Day 3 | CTR_HE_ | VitC_DW_ | 7 | 8 | 0.000873 | *** | 0.01746 | * |
| Day 3 | CTR_HE_ | VitC_MC_ | 7 | 8 | 0.00965 | ** | 0.042889 | * |
| Day 4 | CTR_HE_ | VitC_DW_ | 7 | 7 | 0.00515 | ** | 0.034333 | * |
| Day 4 | CTR_HE_ | VitC_MC_ | 7 | 7 | 0.00814 | ** | 0.0407 | * |
| Day 6 | CTR_HE_ | VitC_MC_ | 8 | 6 | 0.00305 | ** | 0.0244 | * |
| Day 7 | CTR_HE_ | VitC_MC_ | 6 | 5 | 0.0024 | ** | 0.024 | * |
| Day 7 | CTR_HE_ | VitC_PO_ | 6 | 5 | 0.000165 | *** | 0.0066 | ** |
| Day 7 | VitC_DW_ | VitC_PO_ | 3 | 5 | 0.0021 | ** | 0.024 | * |

**Supplementary Figure 4B.**

| Day | group1 | group2 | n1 | n2 | p | p.signif | p.adj | p.adj.signif |
| --- | --- | --- | --- | --- | --- | --- | --- | --- |
| Day 2 | CTR_HE_ | CTR_DSS_ | 4 | 6 | 8.52E-04 | *** | 0.005742 | ** |
| Day 2 | CTR_HE_ | VitC_DW_ | 4 | 7 | 5.42E-03 | ** | 0.019129 | * |
| Day 2 | CTR_HE_ | VitC_MC_ | 4 | 8 | 7.15E-03 | ** | 0.023833 | * |
| Day 2 | CTR_HE_ | VitC_PO_ | 4 | 7 | 9.61E-03 | ** | 0.030347 | * |
| Day 3 | CTR_HE_ | CTR_DSS_ | 7 | 8 | 9.45E-04 | *** | 0.005742 | ** |
| Day 3 | CTR_HE_ | VitC_DW_ | 7 | 8 | 6.04E-05 | **** | 0.000906 | *** |
| Day 3 | CTR_HE_ | VitC_MC_ | 7 | 8 | 5.40E-03 | ** | 0.019129 | * |
| Day 3 | CTR_HE_ | VitC_PO_ | 7 | 8 | 1.26E-02 | * | 0.0378 | * |
| Day 4 | CTR_HE_ | CTR_DSS_ | 7 | 6 | 5.28E-05 | **** | 0.000906 | *** |
| Day 4 | CTR_HE_ | VitC_DW_ | 7 | 7 | 7.99E-06 | **** | 0.000479 | *** |
| Day 4 | CTR_HE_ | VitC_MC_ | 7 | 7 | 8.85E-04 | *** | 0.005742 | ** |
| Day 4 | CTR_HE_ | VitC_PO_ | 7 | 6 | 5.39E-03 | ** | 0.019129 | * |
| Day 5 | CTR_HE_ | VitC_DW_ | 4 | 6 | 4.93E-04 | *** | 0.00493 | ** |
| Day 5 | CTR_HE_ | VitC_MC_ | 4 | 5 | 5.09E-03 | ** | 0.019129 | * |
| Day 5 | CTR_HE_ | VitC_PO_ | 4 | 5 | 1.86E-03 | ** | 0.0093 | ** |
| Day 6 | CTR_HE_ | CTR_DSS_ | 8 | 8 | 1.11E-03 | ** | 0.006055 | ** |
| Day 6 | CTR_HE_ | VitC_MC_ | 8 | 6 | 2.64E-05 | **** | 0.000792 | *** |
| Day 6 | CTR_HE_ | VitC_PO_ | 8 | 6 | 4.99E-03 | ** | 0.019129 | * |
| Day 7 | CTR_HE_ | VitC_MC_ | 6 | 5 | 3.74E-04 | *** | 0.004488 | ** |
| Day 7 | CTR_HE_ | VitC_PO_ | 6 | 5 | 9.57E-04 | *** | 0.005742 | ** |
